# Supplementary material for: Synthesis and Photophysical Properties of α-(N-Biphenyl)-Substituted 2,2′-Bipyridine-Based Push–Pull Fluorophores
Source: Molecules. 2022 Oct 13;27(20):6879. doi: 10.3390/molecules27206879 (PMC9608819; doi:10.3390/molecules27206879)
Supplement: Supplementary file 1 [file molecules-27-06879-s001.zip › molecules-1959037-supplementary.pdf]

Synthesis and Photophysical Properties of α-(*N*-Biphenyl)-Substituted 2,2'-Bipyridine-Based Push–Pull Fluorophores

Ekaterina S. Starnovskaya [1,2], Dmitry S. Kopchuk [1,2], Albert F. Khasanov [1], Olga S. Taniya [1], Igor L. Nikonov [1,2], Maria I. Valieva [1,2], Dmitry E. Pavlyuk [1], Alexander S. Novikov [3,4], Grigory V. Zyryanov [1,2,]* and Oleg N. Chupakhin [1,2]

[1] Chemical Engineering Institute, Ural Federal University, 19 Mira St., Yekaterinburg 620002, Russia

[2] I. Ya. Postovsky Institute of Organic Synthesis of RAS (Ural Division), 22/20 S. Kovalevskoy/Akademicheskaya St., Yekaterinburg 62099, Russia

[3] Institute of Chemistry, Saint Petersburg State University, 7/9 Universitetskaya Nab., Saint Petersburg 199034, Russia

[4] Research Institute of Chemistry, Peoples' Friendship University of Russia (RUDN University), 6 Miklukho-Maklaya St., Moscow 117198, Russia

SUPPLEMENTARY MATERIALS

Contents

1. Materials and equipment ... 4
2. Experimental procedures. ... 4
3. Table S1. Selected bond lengths and angles for compound 3a. ... 10
4. Figure S1. ^{1}H NMR (400 MHz, DMSO-d_6) of 5a ... 11
5. Figure S2. ^{1}H NMR (400 MHz, DMSO-d_6) of 5b ... 12
6. Figure S3. ^{1}H NMR (400 MHz, DMSO-d_6) of 5c ... 13
7. Figure S4. ^{1}H NMR (400 MHz, DMSO-d_6) of 6a ... 14
8. Figure S5. ^{1}H NMR (400 MHz, DMSO-d_6) of 6b ... 15
9. Figure S6. ^{1}H NMR (400 MHz, DMSO-d_6) of 6c ... 16
10. Figure S7. ^{1}H NMR (400 MHz, DMSO-d_6) of 3a ... 17
11. Figure S8. ^{13}C NMR (400 MHz, DMSO-d_6) of 3a ... 18
12. Figure S9.^{1}H NMR (400 MHz, DMSO-d_6) of 3b ... 19
13. Figure S10.^{13}C NMR (400 MHz, DMSO-d_6) of 3b ... 20
14. Figure S11.^{1}H NMR (400 MHz, DMSO-d_6) of 3c ... 21
15. Figure S12.^{13}C NMR (400 MHz, DMSO-d_6) of 3c ... 22
16. Figure S13.^{1}H NMR (400 MHz, DMSO-d_6) of 3d ... 23
17. Figure S14.^{13}C NMR (400 MHz, DMSO-d_6) of 3d ... 24
18. Figure S15.^{1}H NMR (400 MHz, DMSO-d_6) of 3e ... 25
19. Figure S16.^{13}C NMR (400 MHz, DMSO-d_6) of 3e ... 26
20. Figure S17.^{1}H NMR (400 MHz, DMSO-d_6) of 3f ... 27
21. Figure S18.^{13}C NMR (400 MHz, DMSO-d_6) of 3f ... 28
22. Figure S19.^{1}H NMR (400 MHz, DMSO-d_6) of 3g ... 29
23. Figure S20.^{13}C NMR (400 MHz, DMSO-d_6) of 3g ... 30
24. Figure S21.^{1}H NMR (400 MHz, DMSO-d_6) of 3h ... 31
25. Figure S22.^{13}C NMR (400 MHz, DMSO-d_6) of 3h ... 32
26. Figure S23.^{1}H NMR (400 MHz, DMSO-d_6) of 3i ... 33
27. Figure S24.^{13}C NMR (400 MHz, DMSO-d_6) of 3i ... 34
28. Figure S25.^{1}H NMR (400 MHz, DMSO-d_6) of 3j ... 35
29. Figure S26.^{13}C NMR (400 MHz, DMSO-d_6) of 3j ... 36
30. Figure S27.^{1}H NMR (400 MHz, DMSO-d_6) of 3k ... 37
31. Figure S28.^{13}C NMR (400 MHz, DMSO-d_6) of 3k ... 38
32. Figure S29.^{1}H NMR (400 MHz, DMSO-d_6) of 3l ... 39
33. Figure S30.^{13}C NMR (400 MHz, DMSO-d_6) of 3l ... 40
34. Figure S31. Absorption and emission spectra of 3a-l and 5a-c in THF ... 41
35. Table S2 Orientation polarizability for solvents (Δf), absorption and fluorescence emission maxima ($\lambda_{abs,}$ λ_{em}, nm) and Stokes shift (nm, cm^{-1}) of 3b in different solvents ... 44

36. Figure S32 Normalized fluorescence emission spectra of 3b in different solvents 44

37. Table S3 Orientation polarizability for solvents (Δf), absorption and fluorescence emission maxima ($\lambda_{abs,}$ λ_{em}, nm) and Stokes shift (nm, cm^{-1}) of 3g in different solvents 45

38. Figure S33 Normalized fluorescence emission spectra of 3g in different solvents 45

39. Table S4 Orientation polarizability for solvents (Δf), absorption and fluorescence emission maxima ($\lambda_{abs,}$ λ_{em}, nm) and Stokes shift (nm, cm^{-1}) of 3i in different solvents 46

40. Figure S34 Normalized fluorescence emission spectra of 3i in different solvents 46

41. Table S5 Orientation polarizability for solvents (Δf), absorption and fluorescence emission maxima ($\lambda_{abs,}$ λ_{em}, nm) and Stokes shift (nm, cm^{-1}) of 3l in different solvents 47

42. Figure S35 Normalized fluorescence emission spectra of 3l in different solvents 47

43. Table S6. Data for the Lippert-Mataga plots of 3b,g,i,l 48

44. Table S7. Data for the Lippert-Mataga plots of 3b,g,i,l in the range of polar solvents (DMSO, DMF, acetonitrile, methanol) 48

45. Table S8. Data for fluorescence emission maximum relationship with the empirical solvent polarity parameter $E_T(30)$ plots 48

46. Figure S36. Difference in dipole moments ($\Delta\mu_{)}$ of some 2,2'-bipyridine derivatives and analogs calculated according to Lippert-Mataga equation. 49

47. Figure S37. Plot of the Hammett constants at the meta (a) and para (b) position versus the values of the fluorophore emission maxima 50

48. Table S9. Molecular electrostatic potential energy map and calculated dipole moments (in Debye) in ground (S_0) and excited (S_1) multiplicity states for 3b,g,i,l. 50

49. Table S10. HOMO-LUMO Energy levels for fluorophores 3b,g,i,l in a gas phase 51

50. Figure S38. Tauc Plots for 3b,g,i,l constructed based on UV/Vis data 52

References .... **Error! Bookmark not defined.**

1. **Materials and equipment.**

Unless otherwise indicated, all common reagents and solvents were used from commercial suppliers without further purification. 6-Phenyl-3-(pyridin-2-yl)-1,2,4-triazine-5-carbonitrile **2a** and 6-(4-tolyl)-3-(pyridin-2-yl)-1,2,4-triazine-5-carbonitrile **2b** were synthesized according reported literature [1].

Melting points were determined on Boetius combined heating stages. TLC and column chromatography were carried out on SiO_2. ^{1}H NMR and ^{13}C NMR spectra were recorded at room temperature at 400 and 100 MHz respectively, on a Bruker DRX-400 spectrometer using $CDCl_3$ or DMSO-d_6 as the solvent. Hydrogen chemical shifts were referenced to the hydrogen resonance of the corresponding solvent (DMSO-d_6, δ = 2.50 ppm or $CDCl_3$, δ = 7.26 ppm). Carbon chemical shifts were referenced to the carbon resonances of the solvent ($CDCl_3$, δ = 77.16 ppm). Peaks were labeled as singlet (s), doublet (d), triplet (t), doublet of doublets (dd), doublet of doublets of doublets (ddd) and multiplet (m). Mass-spectra were recorded on MicrOTOF-Q II (Bruker Daltonics), electrospray as a method of ionization UV–vis absorption spectra were recorded on the Shimadzu UV-1800 spectrophotometer, and emission spectra were measured on the Horiba FluoroMax-4 by using quartz cells with 1 cm path length at room temperature. Absolute quantum yields of luminescence of target compounds in solution were measured by using the Integrating Sphere Quanta-φ of the Horiba-Fluoromax-4 at room temperature. The fluorometric titration was performed by the single-point methodology using Horiba FluoroMax 4

2. **Experimental procedures.**

*General method for the synthesis of 5-arylamino-1,2,4-triazines **1a-c** or **5a-c***

The mixture of corresponding 5-cyano-1,2,4-triazine **2a,b** (1 mmol) and corresponding aniline (1.05 mmol) was stirred at 200 °C for 8 h under argon atmosphere. The products were used in the next step without additional purification. Analytical samples were obtained by flash chromatography with chloroform as an eluent.

N-(4-bromophenyl)-6-phenyl-3-(pyridin-2-yl)-1,2,4-triazin-5-amine (5a). Yield 386 mg**,** (0.96 mmol), 87%. ^{1}H NMR (400 MHz, DMSO-d_6): δ 7.43-7.63 (m, 7H, Ph), 7.75-7.90 (m, 3H, 7.75-7.90, Ph, H-5(Py)), 7.94 (ddd, J = 7.6 Hz, J = 7.6 Hz, J = 1.6Hz, 1H, H-4(Py)), 8.30-8.37 (m, 1H, H-3(Py)), 8.76 (d, J = 4Hz, H-6(Py)), 9.20 (s, 1H, NH).

N-(4-bromophenyl)-6-(p-tolyl)-3-(pyridin-2-yl)-1,2,4-triazin-5-amine (5b). Yield 390 mg**, (**0.88 mmol), 92%. ^{1}H NMR (400 MHz, DMSO-d_6): δ 2.48 (s, 3H, CH_3), 7.39 (d, J = 8.0 Hz, 2H, Ph), 7.44 7.53 (m, 3H, pH, H-5(Py)), 7.74 (d, J = 8.0 Hz, 2H, Ph), 7.84-7.96 (m, 3H, Ph, H-4(Py)), 8.36 (d, J = 8.0Hz, 1H, H-3(Py)), 8.76 (d, J = 4.4 Hz, 1H, H-6(Py)), 9.10 (s, 1H, NH)

N-(3-bromophenyl)-6-(p-tolyl)-3-(pyridin-2-yl)-1,2,4-triazin-5-amine (5c). Yield 375 mg (90 mmol), 90%. ^{1}H NMR (400 MHz, DMSO-d_6): δ 2.49 (s, 3H, CH_3), 7.18-7.30 (m, 3H, Ph), 7.39 (d, 7.6 Hz, 2H, Ph), 7.46-7.52 (m, 1H, H-5(Py)), 7.73 (d, J = 7.6 Hz, 2H, Ph), 7.94 (ddd, J = 7.6 Hz, J = 7.6 Hz,

J = 1.6 Hz, 1H, H-4(Py)), 7.99 (d, J = 8.4 Hz, 1H), 8.19 (s, 1H, Ph), 8.37 (d, J = 8.0 Hz, H-3(Py)), 8.78 (d, J = 4.8 Hz, H-6(Py)), 9.14 (s, 1H, NH)

*General method for the synthesis of N-aryl-1-(pyridine-2-yl)-6,7-dihydro-5H-cyclopenta[c]pyridin-3-amines **3a-l** or **6a-c**.*

The mixture of corresponding 5-arylamino-1,2,4-triazine **1a-c** (0.3 mmol) or **5a-c** and 1-morpholinocyclopentene (1.5 mmol) was stirred at 200°C for 2 h under argon atmosphere. Then, the additional portion of 1-morpholinocyclopentene (0.75 mmol) was added and the resulting mixture was stirred for additional 1 h at the same conditions. The products were separated by flash chromatography (DCM as eluent) and then were purified by recrystallization (ethanol).

N-(4-bromophenyl)-4-phenyl-1-(pyridin-2-yl)-6,7-dihydro-5H-cyclopenta[c]pyridin-3-amine (6a). Yield 89 mg (0.20mmol), 68%. ^{1}**H NMR** (400 MHz, DMSO-*d*$_6$): δ 2.01 (m, 2H, CH_2-6), 2.68 (t, 2H, 7.9 Hz, CH_2-7), 3.42 (t, 2H, J = 7.9 Hz, CH_2-5), 6.97 (s, 1H, NH), 7.27-7.34 (m, 3H, Ph), 7.37-7.45 (m, 3H, Ph, H-5(Py)), 7.47-7.56 (m, 4H, Ph), 7.85 (ddd, J = 7.6 Hz, J = 7.6 Hz, J = 1.6 Hz, 1H, H-4(Py)), 8.23 (J = d, 8.0 Hz, 1H, H-3(Py)), 8.64 (J = d, 4.0 Hz, 1H, H-6(Py)).

N-(4-bromophenyl)-4-(p-tolyl)-1-(pyridin-2-yl)-6,7-dihydro-5H-cyclopenta[c]pyridin-3-amine (6b). Yield 110 mg (0.24 mmol), 72%. ^{1}**H NMR** (400 MHz, DMSO-*d*$_6$): δ 2.00 (m, 2H, CH_2-6), 2.44 (s, 3H, Me), 2.67 (t, 2H, J = 7.9 Hz, CH_2-7), 3.41 (t, 2H, J = 7.9 Hz, CH_2-5), 6.94 (s, 1H, NH), 7.25-7.35 (m, 7H, Ph, H-5(Py)), 7.51-7.58 (m, 2H, Ph), 7.85 (ddd, J = 8.0 Hz, J = 8.0 Hz, J = 1.2 Hz, 1H, H-4(Py)), 8.27 (d, 8.0 Hz, 1H, H-3(Py)), 8.63 (d, 4.0 Hz, H-6(Py))

N-(3-bromophenyl)-4-(p-tolyl)-1-(pyridin-2-yl)-6,7-dihydro-5H-cyclopenta[c]pyridin-3-amine (6c). Yield 68 mg (0.77 mmol), 70%. ^{1}**H NMR** (400 MHz, DMSO-*d*$_6$): δ δ 2.01 (m, 2H, CH_2-6), 2.45 (s, 3H, Me), 2.67 (t, 2H, J = 7.9 Hz, CH_2-7), 3.43 (t, 2H, J = 7.9 Hz, CH_2-5), 6.96 (dd, J = 1.2 Hz, J = 8.0Hz, 1H, Ph), 7.02 (s, 1H, NH), 7.10 (t, J = 8.0 Hz, 1H, Ph), 7.25-7.35 (m, 5H, Ph, H-5(Py)), 7.41 (dd, J = 1.2 Hz, J = 8.0 Hz, 1H, Ph), 7.84 (ddd, J = 8.0 Hz, J = 8.0 Hz, J = 1.6 Hz, 1H, H-4(Py)), 8.07 (t, J = 2.0 Hz, 1H, Ph), 8.29 (d, J = 8.0 Hz, 1H, H-3(Py)), 8.64 (d, J = 4.0 Hz, H-6(Py))

General method for the synthesis N-aryl-1-(pyridine-2-yl)-6,7-dihydro-5H-cyclopenta[c]pyridin-3-amines 3a-l via Suzuki cross-coupling reaction

The corresponding compound **6a-c** (0.3 mmol) was dissolved in THF (15 mL), followed by addition of the corresponding boronic acid (0.33 mmol), $Pd(tpp)_2Cl_2$ (6.3 mg, 0.009 mmol), triphenylphosphine (3.9 mg, 0.015 mmol), solution of K_2CO_3 (415 mg, 3 mmol) in water (15 mL). The resulting mixture was stirred at 90°C in argon atmosphere for 24 h. After completion, the reaction mixture was extracted with ethyl acetate (10 mL). The organic phase was washed with aqueous solution of KOH, ammonium chloride and water, and dried with anhydrous sodium sulfate. The solvent was removed under

reduced pressure. Ethanol was added to the residue and the obtained precipitate was filtered off, washed with ethanol and dried.

N-([1,1'-biphenyl]-3-yl)-4-phenyl-1-(pyridin-2-yl)-6,7-dihydro-5H-cyclopenta[c]pyridin-3-amine (3a) Yield 98 mg (0.23 mmol), 74% **^{1}H NMR** (400 MHz, DMSO-*d$_6$*): δ 2.02 (m, 2H, CH_2-6), 2.67 (t, 2H, J = 7.9 Hz, CH_2-7), 3.44 (t, 2H, J = 7.9 Hz, CH_2-5), 6.88 (s, 1H, NH), 7.11 (d, J = 8.0Hz, 1H, Ph), 7.24-7.33 (m, 3H, Ph), 7.36-7.48 (m, 6H, Ph, H-5(Py)), 7.51-7.57 (m, 2H, Ph), 7.58-7.63 (m, 2H, Ph), 7.77 (ddd, J = 7.6Hz, J = 7.6Hz, J = 1.6Hz, H-4(Py)), 8.13 (t, J = 1.6 Hz, Ph), 8.33 (d, J = 8.0 Hz, H-3(Py)), 8.65 (d, 4Hz, H-6(Py)). **^{13}C NMR** (100 MHz, $CDCl_3$) δ 158.7, 155.8, 150.3, 148.5, 141.8, 141.8, 141.6, 136.2, 136.0, 131.0, 129.6, 129.5, 129.00, 128.6, 128.2, 127.2, 127.2, 123.0, 122.4, 120.6, 119.8, 117.3, 117.3, 33.2, 32.4, 25.5. **ESI-MS**, m/z: required: 440.20 $[M+H]^+$, found 440.20 $[M+H]^+$. Elemental analyses for CHN: Found C, 84.68; H, 5.72; N, 9.58%; molecular formula $C_{31}H_{25}N_3$ requires C, 84.71; H, 5.73; N, 9.56%.

$N^{4'}$,$N^{4'}$-diphenyl-N^{3}-(4-phenyl-1-(pyridin-2-yl)-6,7-dihydro-5H-cyclopenta[c]pyridin-3-yl)-[1,1'-biphenyl]-3,4'-diamine (3b) Yield 92 mg (0.15 mmol), 67%. ^{1}H NMR (400 MHz, DMSO-*d$_6$*): δ 2.02 (m, 2H, CH_2-6), 2.46 (s, 3H, CH_3), 2.68 (t, 2H, J = 7.6 Hz, CH_2-7), 3.42 (t, 2H, J = 7.6 Hz, CH_2-5), 6.81 (s, 1H, NH), 7.00 (m, 2H, Ph), 7.04-7.12 (m, 6H, Ph), 7.20-7.29 (m, 5H, Ph), 7.29-7.41 (m, 5H, Ph, H-5(Py)), 7.50 (d, J = 8.8Hz, 2H, Ph), 7.70 (ddd, J = 7.2 Hz, J = 7.2 Hz, J = 0.8 Hz, 1H, H-4(Py)), 8.10-8.14 (m, 3H, Ph), 8.32 (d, J = 8.0 Hz, H-3(Py)), 8.63 (d, J = 4Hz, H-6(Py)). **^{13}C NMR** (100 MHz, $CDCl_3$) δ 158.8, 155.8, 150.5, 148.4, 147.8, 147.3, 147.0, 141.9, 141.3, 138.0, 136.1, 135.9, 132.8, 131.0, 130.2, 129.5, 129.3, 129.0, 128.0, 124.3, 124.1, 123.0, 122.8, 122.3, 120.7, 119.2, 116.8, 33.2, 32.4, 25.5, 21.4. **ESI-MS**, m/z: required: 621.29 $[M+H]^+$, calculated 621.29 $[M+H]^+$. Elemental analyses for CHN: Found C, 85.16; H, 5.82; N, 9.01%; molecular formula $C_{44}H_{36}N_4$ requires C, 85.13; H, 5.85; N, 9.03%.

4-Phenyl-1-(pyridin-2-yl)-N-(3',4',5'-trimethoxy-[1,1'-biphenyl]-3-yl)-6,7-dihydro-5H-cyclopenta[c]pyridin-3-amine (3c) Yield 79 mg (0.15 mmol), 69% **^{1}H NMR** (400 MHz, DMSO-*d$_6$*): δ 2.01 (m, 2H, CH_2-6), 2.45 (s, 3H, CH_3), 2.68 (t, 2H, J = 7.6 Hz, CH_2-7), 3.43 (t, 2H, J = 7.6 Hz, CH_2-5), 3.73 (s, 3H, CH_3O-), 3.78 (s, 6H, CH_3O-), 6.78 (s, 2H, Ph), 6.82 (s, 1H, NH), 7.07 (d, J = 8.0 Hz, 1H, Ph), 7.21-7.42 (m, 7H, Ph, H-5(Py)), 7.73 (ddd, J = 7.6 Hz, J = 7.6 Hz, J = 1.6 Hz, 1H, H-4(Py)), 8.02 (s, 1H, Ph), 7.27 (d, J = 8.0 Hz, 1H, H-3(Py)), 8.63 (d, J = 3.6 Hz, H-6(Py)). **^{13}C NMR** (100 MHz, $CDCl_3$) δ 158.8, 155.9, 153.3, 150.5, 148.5, 147.3, 142.0, 141.9, 138.0, 137.7, 137.6, 136.3, 132.8, 131.2, 130.2, 129.4, 129.0, 122.8, 122.3, 120.8, 119.6, 117.2, 117.1, 104.6, 61.0, 56.2, 33.2, 32.4, 25.5, 21.4. **ESI-MS**, m/z: required: 544.25 $[M+H]^+$, found 544.25 $[M+H]^+$. Elemental analyses for CHN: Found C, 77.29; H, 6.15; N, 7.74%; molecular formula $C_{35}H_{23}N_3O_3$ requires C, 77.32; H, 6.12; N, 7.73%.

N-(4'-methoxy-[1,1'-biphenyl]-3-yl)-4-phenyl-1-(pyridin-2-yl)-6,7-dihydro-5H-cyclopenta[c]pyridin-3-amine (3d) Yield 89 mg (0.19 mmol), 72%. **^{1}H NMR** (400 MHz, DMSO-*d$_6$*): δ 2.02 (m, 2H, CH_2-6), 2.46 (s, 3H, CH_3), 2.68 (t, 2H, 7.6 Hz, CH_2-7), 3.43 (t, 2H, 7.6 Hz, CH_2-5), 3.83 (s,

3H, CH_3O-), 6.79 (s, 1H, NH), 6.93 (d, J = 8.8 Hz, 2H, Ph), 7.06 (d, J = 7.6 Hz, 1H, Ph), 7.23 (t, J = 8.0 Hz, J = 8.0 Hz, 1H, Ph), 7.27-7.37 (m, 6H, Ph, H-5(Py)), 7.53 (d, J = 8.8 Hz, 2H Ph), 7.80 (ddd, J = 7.6 Hz, 7.6 Hz, 1.6 Hz, 1H, H-4(Py)), 8.06 (t, 1.6 Hz, 1H, Ph), 8.33 (d, J = 8.0 Hz, 1H, H-3(Py)), 8.64 (d, J = 4.0 Hz, 1H, H-6(Py)). **^{13}C NMR** (100 MHz, $CDCl_3$) δ 159.1, 158.9, 155.8, 150.5, 148.5, 147.3, 141.9, 141.4, 138.0, 136.1, 134.2, 132.9, 131.0, 130.2, 129.5, 129.0, 128.2, 123.0, 122.3, 120.6, 119.3, 116.8, 116.7, 114.0, 55.4, 33.2, 32.4, 25.5, 21.4. **ESI-MS**, m/z: required: 484.23 $[M+H]^+$, found 484.23 $[M+H]^+$. Elemental analyses for CHN: Found C, 82.01; H, 6.02; N, 8.73%; molecular formula $C_{33}H_{29}N_3O$ requires C, 81.96; H, 6.04; N, 8.69%.

N-(2',5'-dimethoxy-[1,1'-biphenyl]-3-yl)-4-phenyl-1-(pyridin-2-yl)-6,7-dihydro-5H-cyclopenta[c]pyridin-3-amine (3e) Yield 108 mg (0.24 mmol), 72%. **^{1}H NMR** (400 MHz, DMSO-d_6): δ 2.01 (m, 2H, CH_2-6), 2.68 (t, 2H, J = 7.6 Hz, CH_2-7), 3.43 (t, 2H, J = 7.6 Hz, CH_2-5), 3.68 (s, 3H, CH_3O-), 3.74 (s, 3H, CH_3O-), 6.75 (s, 1H, NH), 6.78-6.73 (m, 2H, Ph), 6.83-6.87 (m, 1H, Ph), 6.89-6.94 (m, 1H, Ph), 6.99 (d, J = 7.6Hz, 1H, Ph), 7.21 (t, J = 8.0 Hz, 1H, Ph), 6.26 (dd, J = 5.6 Hz, J = 1.6 Hz, 1H, H-5(Py)), 7.37-7.47 (m, 3H, Ph), 7.50-7.57 (m, 2H, Ph), 7.74 (ddd, J = 7.6 Hz, J = 7.6 Hz, J = 1.6 Hz, 1H. H-4(Py)), 7.84 (s, 1H, Ph), 8.28 (d, J = 8.0 Hz, H-3(Py)), 8.62 (d, J = 4.0 Hz, 1H, H-6(Py)). **^{13}C NMR** (100 MHz, $CDCl_3$) δ 153.8, 150.9, 148.3, 131.0, 129.6, 129.4, 128.2, 116.9, 113.0, 112.8, 56.5, 55.8, 33.1, 32.4, 29.7, 29.7, 25.5. **ESI-MS**, m/z: required: 500.23 $[M+H]^+$, found 500.23 $[M+H]^+$. Elemental analyses for CHN: Found C, 79.37; H, 5.89; N, 8.42%; molecular formula $C_{33}H_{29}N_3O_2$ requires C, 79.33; H, 5.85; N, 8.41%.

N-(2',5'-dimethyl-[1,1'-biphenyl]-3-yl)-4-phenyl-1-(pyridin-2-yl)-6,7-dihydro-5H-cyclopenta[c]pyridin-3-amine (3f) Yield 88 mg (0.18 mmol), 73%. **^{1}H NMR** (400 MHz, DMSO-d_6): δ 2.02 (m, 2H, CH_2-6), 2.20 (s, 1H, CH_3), 2.31 (s, 1H, CH_3), 2.68 (t, 2H, J = 7.6 Hz, CH_2-7), 3.42 (t, 2H, J = 7.6 Hz, CH_2-5), 6.78 (d, 7.6 Hz, 2H, Ph), 6.95-7.02 (m ,2H, Ph, NH), 7.07 (d, J = 7.6 Hz, 1H, Ph), 7.19-7.29 (m, 2H, Ph, H-5(Py)), 7.39-7.47 (m, 4H, Ph), 7.49-7.56 (m, 2H, Ph), 7.59 (s, 1H, Ph), 7.70 (t, J = 8.0 Hz, 1H, H-4(Py)), 8.23 (d, J = 8.0 Hz, 1H, H-3(Py)), 8.62 (d, J = 4.0 Hz, 1H, H-6(Py)). **^{13}C NMR** (100 MHz, $CDCl_3$) 150.4, 148.4, 142.8, 142.0, 141.0, 136.3, 135.9, 134.9, 132.2, 131.1, 130.4, 130.1, 129.6, 129.4, 128.3, 128.2, 127.8, 123.0, 122.4, 122.2, 120.8, 119.8, 116.9, 33.1, 32.4, 25.5, 21.0, 20.0. **ESI-MS**, m/z: required: 468.24 $[M+H]^+$, found 468.24 $[M+H]^+$. Elemental analyses for CHN: Found C, 84.73; H, 6.30; N, 8.97%; molecular formula $C_{33}H_{29}N_3$ requires C, 84.76; H, 6.25; N, 8.99%.

$N^{4'}$,$N^{4'}$-diethyl-N^3-(4-phenyl-1-(pyridin-2-yl)-6,7-dihydro-5H-cyclopenta[c]pyridin-3-yl)-[1,1'-biphenyl]-3,4'-diamine (3g) Yield 67 mg **(**0.13 mmol), 63%**.** **^{1}H NMR** (400 MHz, DMSO-d_6): δ 1.17 (t, 7.6 Hz, 6H, $\underline{CH_3}CH_2$-), 2.00 (m, 2H, CH_2-6), 2.45 (s, 3H, CH_3-), 2.68 (t, J = 7.6 Hz, 2H, CH_2-7), 3.34-3.46 (m, 6H, $CH_3\underline{CH_2}$-, CH_2-5), 6.67 (d, J = 7.2 Hz, 2H, Ph), 6.89 (s, 1H, NH), 7.05 (d, J = 6.8 Hz, 1H, Ph), 7.213 (t, J = 7.6 Hz, Ph), 7.27-7.37 (m, 6H, Ph, H-5(Py))), 7.37-7.48 (m, 2H, Ph), 7.82 (ddd, J = 7.6 Hz, J = 7.6 Hz, J = 1.6 Hz, H-4(Py)), 7.99 (s, 1H, Ph), 8.32 (d, J = 8.0 Hz, H-3(Py)), 8.66 (d, J = 3.6

Hz, 1H, H-6(Py)). **^{13}C NMR** (100 MHz, $CDCl_3$): 159.0, 155.7, 150.6, 148.4, 147.3, 147.1, 141.9, 141.8, 137.9, 136.2, 132.9, 130.8, 130.1, 129.5, 128.8, 128.0, 123.0, 122.2, 120.6, 118.9, 116.3, 115.9, 112.0, 44.5, 33.2, 32.4, 25.5, 21.4, 12.7. **ESI-MS**, m/z: required: 525.29 $[M+H]^+$, found 525.29 $[M+H]^+$. Elemental analyses for CHN: Found C, 85.14; H, 5.79; N, 9.04%; molecular formula $C_{44}H_{36}N_4$ requires C, 85.13; H, 5.85; N, 9.03%.

N-([1,1'-biphenyl]-4-yl)-4-phenyl-1-(pyridin-2-yl)-6,7-dihydro-5H-cyclopenta[c]pyridin-3-amine (3h) Yield 0.70 mg (70 mmol). 70%. ^{1}H NMR (400 MHz, DMSO-d_6): δ 7.04 (s, 1H, NH), 7.25 (t, 7.6 Hz, 1H, Ph), 7.29-7.48 (m, 6H, Ph, H-5(Py)), 7.48-7.62 (m, 6H, Ph), 7.65 (d, J = 8.8 Hz, 2H, Ph), 7.91 (t, J = 8.0 Hz, H-4(Py)), 8.30 (d, J = 8.0 Hz, H-3(Py)), 8.66 (d, J = 4.8 Hz, H-6(Py)). **^{13}C NMR** (100 MHz, $CDCl_3$): δ 148.2, 141.0, 131.3, 130.6, 129.5, 129.3, 128.7, 128.3, 127.7, 127.3, 126.6, 126.6, 32.7, 32.6, 25.5. **ESI-MS**, m/z: required: 440.20 $[M+H]^+$, found 440.20 $[M+H]^+$. Elemental analyses for CHN: Found C, 84.72; H, 5.72; N, 9.55%; molecular formula $C_{31}H_{25}N_3$ requires C, 84.71; H, 5.73; N, 9.56%.

4-phenyl-1-(pyridin-2-yl)-N-(3',4',5'-trimethoxy-[1,1'-biphenyl]-4-yl)-6,7-dihydro-5H-cyclopenta[c]pyridin-3-amine (3i) Yield 81 mg (0.15 mmol), 67%. **^{1}H NMR** (400 MHz, DMSO-d_6): δ 2.02 (m, 2H, CH_2-6), 2.46 (s, 1H, CH_3), 2.68 (t, 2H, J = 7.6 Hz, CH_2-7), 3.43 (t, 2H, J = 7.6 Hz, CH_2-5), 3.72 (s, 3H, CH_3O-), 3.88 (s, 6H, CH_3O-), 6.78 (d, J = 7.6 Hz, 2H, Ph), 6.82 (s, 1H, NH), 7.27-7.37 (m, 5H, Ph, H-5(Py)), 7.46 (d, J = 8.8Hz, 2H, Ph), 7.64 (d, J = 8.8Hz, 2H, Ph), 7.88 (ddd, J = 7.6 Hz, J = 7.6 Hz, J = 1.6 Hz, 1H, H-4(Py)), 8.31 (d, J = 8.0 Hz, 1H, H-3(Py)), 8.64 (d, J = 5.6 Hz, 1H, H-6(Py)). **^{13}C NMR** (100 MHz, $CDCl_3$) 128.6, 156.0, 153.5, 150.3, 148.4, 147.3, 140.9, 138.0, 137.2, 137.2, 136.4 133.8, 132.8, 131.2, 130.2, 129.5, 127.3, 122.9, 122.4, 120.8, 118.6, 104.0, 61.0, 56.2, 33.2, 32.4, 25.5, 21.4. **ESI-MS**, m/z: required: 544.25 $[M+H]^+$, found 544.25 $[M+H]^+$. Elemental analyses for CHN: Found C, 77.33; H, 6.11; N, 7.73%; molecular formula $C_{35}H_{33}N_3O_3$ requires C, 77.32; H, 6.12; N, 7.73%.

N-(4'-methoxy-[1,1'-biphenyl]-4-yl)-1-(pyridin-2-yl)-4-(p-tolyl)-6,7-dihydro-5H-cyclopenta[c]pyridin-3-amine (3j) Yield 0.72 mg (0.15 mmol), 70% **^{1}H NMR** (400 MHz, DMSO-d_6): δ 2.01 (m, 2H, CH_2-6), 2.46 (s, 1H, CH_3), 2.68 (t, 2H, J = 7.6 Hz, CH_2-7), 3.43 (t, 2H, J = 7.6 Hz, CH_2-5), 3.81 (s, 3H, CH_3O-), 6.76 (s, 1H, NH), 6.91 (d, J = 8.8 Hz, 2H, Ph), 7.26-7.37 (m, 5H, Ph, H-5(Py)), 7.43 (d, J = 8.8 Hz, 2H, Ph), 7.48 (d, J = 8.8 Hz, 2H, Ph), 7.62 (d, J = 8.8 Hz, 2H, Ph), 7.68 (ddd, J = 7.6 Hz, J = 7.6 Hz, J = 1.6 Hz, 1H, H-4(Py)), 8.31 (d, J = 8 Hz, 1H, H-3(Py)), 8.64 (d, J = 4 Hz, 1H, H-6(Py)). **^{13}C NMR** (100 MHz, $CDCl_3$) δ 157.8, 157.6, 154.7, 149.4, 147.4, 146.4, 139.3, 136.9, 135.3. 132.8, 132.4, 131.8, 130.0, 129.1, 128.4, 126.6, 125.9, 121.8, 121.3, 119.6, 117.6, 113.1, 54.3, 32.2, 31.3, 24.5, 20.3. **ESI-MS**, m/z: required: 484.23 $[M+H]^+$, found 484.23 $[M+H]^+$. Elemental analyses for CHN: Found C, 81.98; H, 6.05; N, 8.68%; molecular formula $C_{33}H_{29}N_3O$ requires C, 81.96; H, 6.04; N, 8.69%.

N-(4'-methoxy-[1,1'-biphenyl]-4-yl)-1-(pyridin-2-yl)-4-phenyl-6,7-dihydro-5H-cyclopenta[c]pyridin-3-amine (3k) Yield 0.70 mg **(**0.15 mmol), 66%**. ^{1}H NMR** (400 MHz, DMSO-d_6): δ 2.02 (m, 2H, CH_2-6), 2.68 (t, 2H, J = 7.6 Hz, CH_2-7), 3.42 (t, 2H, J = 7.6 Hz, CH_2-5), 3.81 (s, 3H, CH_3O-

), 6.92 (d, J = 8.8 Hz, 2H, Ph), 7.33 (m, 1H, H-5(Py)), 7.40-7.58 (m, 8H, Ph, NH), 7.61 (d, J = 8.8 Hz, 2H, Ph), 7.89 (ddd, J = 7.6 Hz, J = 7.6 Hz, J = 1.6 Hz, 1H, H-4(Py)), 8.30 (d, J = 8.0 Hz, 1H, H-3(Py)), 8.65 (d, J = 4.0 Hz, 1H, H-6(Py)). **^{13}C NMR** (100 MHz, $CDCl_3$) δ 157.7, 149.3, 147.3, 135.6, 134.8, 132.7, 130.1, 128.6, 128.4, 127.2, 126.6, 125.7, 122.0, 121.5, 119.8, 118.0, 113.1, 54.3, 32.1, 31.4, 24.5. **ESI-MS**, m/z: required: 470.22 $[M+H]^+$, found 470.22 $[M+H]^+$. Elemental analyses for CHN: Found C, 81.86; H, 5.81; N, 8.93%; molecular formula $C_{32}H_{27}N_3O$ requires C, 81.85; H, 5.80; N, 8.95%.

N^4,N^4-diphenyl-$N^{4'}$-(4-phenyl-1-(pyridin-2-yl)-6,7-dihydro-5H-cyclopenta[c]pyridin-3-yl)-[1,1'-biphenyl]-4,4'-diamine (3l). Yield 70 mg (0.12 mmol), 65%. **^{1}H NMR** (400 MHz, DMSO-d_6): δ 2.03 (m, 2H, CH_2-6), 2.70 (t, 2H, J = 7.6 Hz, CH_2-7), 3.44 (t, 2H, J = 7.6 Hz, CH_2-5), 6.97 (t, J = 7.2 Hz, 3H, Ph), 7.03-7.10 (m, 6H, Ph), 7.20-7.27 (m, 4H, Ph), 7.32 (m, 1H, H-5(Py)), 7.41-7.49 (m, 7H, Ph, NH), 7.50-7.58 (m, 2H, Ph), 7.62 (d, J = 8.8 Hz, 2H, Ph), 7.88 (ddd, J = 7.6 Hz, J = 7.6 Hz, J = 1.6 Hz, 1H, H-4(Py)), 8.31 (d, J = 8.0 Hz, 1H, H-3(Py)), 8.66 (d, J = 4.0 Hz, 1H, H-6(Py)). **^{13}C NMR** (100 MHz, $CDCl_3$) δ 149.3, 147.3, 146.8, 145.5, 134.3, 128.6, 130.1, 128.4, 128.2, 127.2, 126.2, 125.8, 123.5, 123.3, 123.2, 122.0, 121.7, 121.6, 118.0, 32.1, 31.4, 24.5. **ESI-MS**, m/z: required: 607.28 $[M+H]^+$, found 607.28 $[M+H]^+$. Elemental analyses for CHN: Found C, 85.10; H, 5.66; N, 9.24%; molecular formula $C_{43}H_{34}N_4$ requires C, 85.12; H, 5.65; N, 9.23%

Crystallography.

The XRD analysis was carried out using equipment of the Center for Joint Use "Spectroscopy and Analysis of Organic Compounds" at the Postovsky Institute of Organic Synthesis of the Russian Academy of Sciences (Ural Branch). The experiments were accomplished on the automated X-ray diffractometer «Xcalibur 3» with CCD detector on standard procedure (MoKα-irradiation, graphite monochromator, ω-scans with 1o step at T= 295(2) K). Empirical absorption correction was applied. The solution and refinement of the structures were accomplished with using Olex program package [2]. The structures were solved by method of the intrinsic phases in ShelXT program and refined by ShelXL by full-matrix least-squared method for non-hydrogen atoms [3]. The H-atoms at C-H bonds were placed in the calculated positions, the H-atoms at N-H bonds were refined independently in isotropic approximation.

Crystal Data for **3a** $C_{31}H_{25}N_3$ (M = 439.54 g/mol): monoclinic, space group $P2_1/n$, a = 9.5548(10) Å, b = 12.3951(12) Å, c = 19.784(3) Å, β = 97.481(10)°, V = 2323.2(5) Å^3, Z = 4, T = 295(2) K, μ(Mo K_α) = 0.074 mm^{-1}, D_{calc} = 1.257 g/cm^3, 11527 reflections measured (7.046° ≤ 2Θ ≤ 54.196°), 5072 unique (R_{int} = 0.0544, R_{sigma} = 0.0999) which were used in all calculations. The final R_1 = 0.0610, wR_2 = 0.1273 (I > 2σ(I)) and R_1 = 0.1587, wR_2 = 0.1841 (all data). Largest diff. peak/hole 0.17/-0.17 ēÅ^{-3}. The result of X-ray diffraction analysis for compound **3a** was deposited with the Cambridge Crystallographic Data Centre (CCDC 2167697).

3. Table S1. Selected bond lengths and angles for compound 3a.

Bond/angles	Å/º	Bond/angles	Å/º
N2 C2	1.392(3)	C2 N2 C21	131.7(2)
N2 C21	1.404(3)	C2 N1 C6	119.6(2)
N1 C2	1.325(3)	C7 N3 C11	117.5(3)
N1 C6	1.361(3)	N1 C2 N2	119.2(2)
N3 C7	1.327(3)	N1C6 C7 N3	178.6(3)
N3 C11	1.342(4)	C2 C3 C15 C16	119.9(3)
C6 C7	1.485(4)	C2 N2 C21 C26	-1.2(5)

4. **Figure S1.** [1]**H NMR (400 MHz, DMSO-*d*$_6$) of 5a**

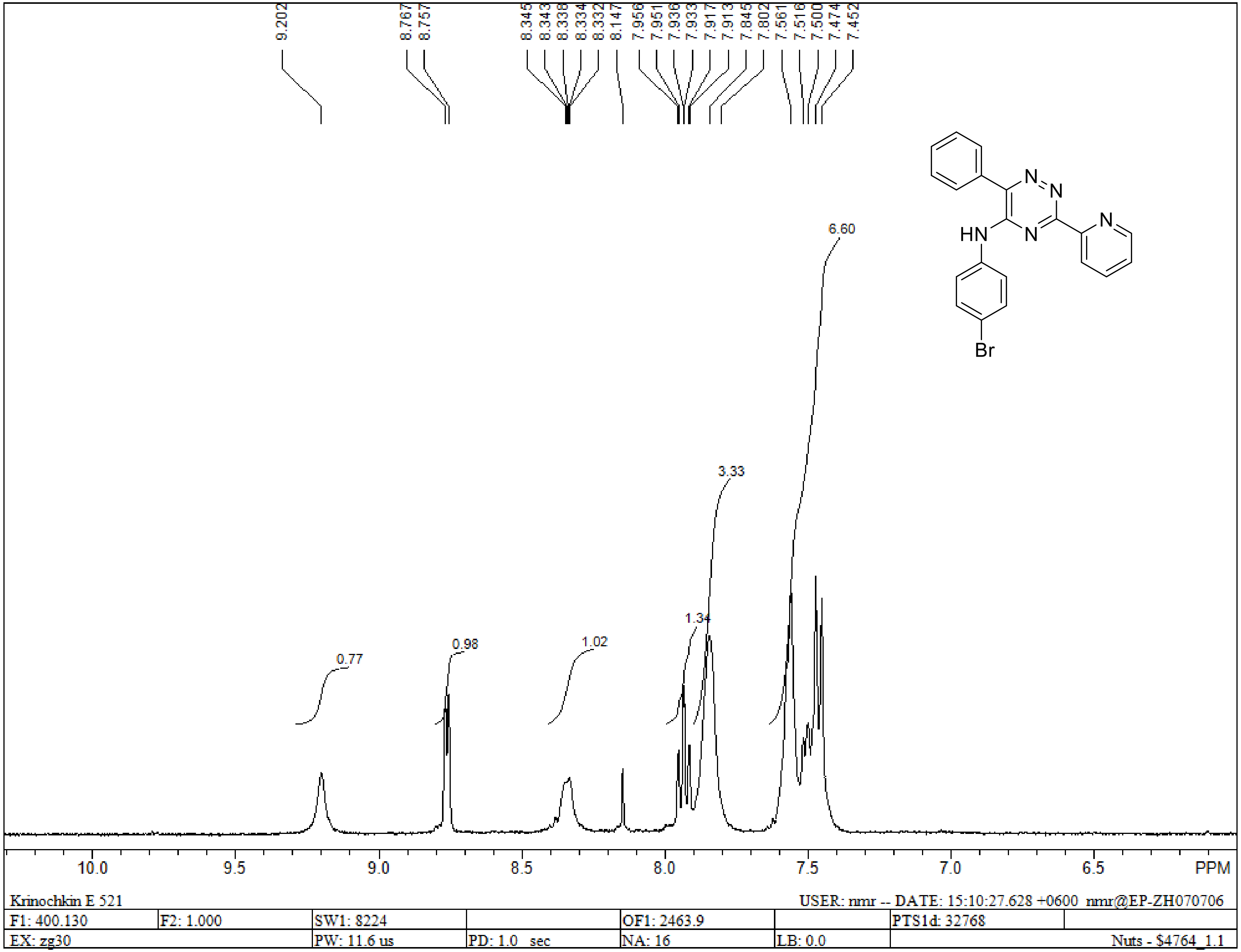

5. **Figure S2.** **^{1}H NMR (400 MHz, DMSO-*d*$_6$) of 5b**

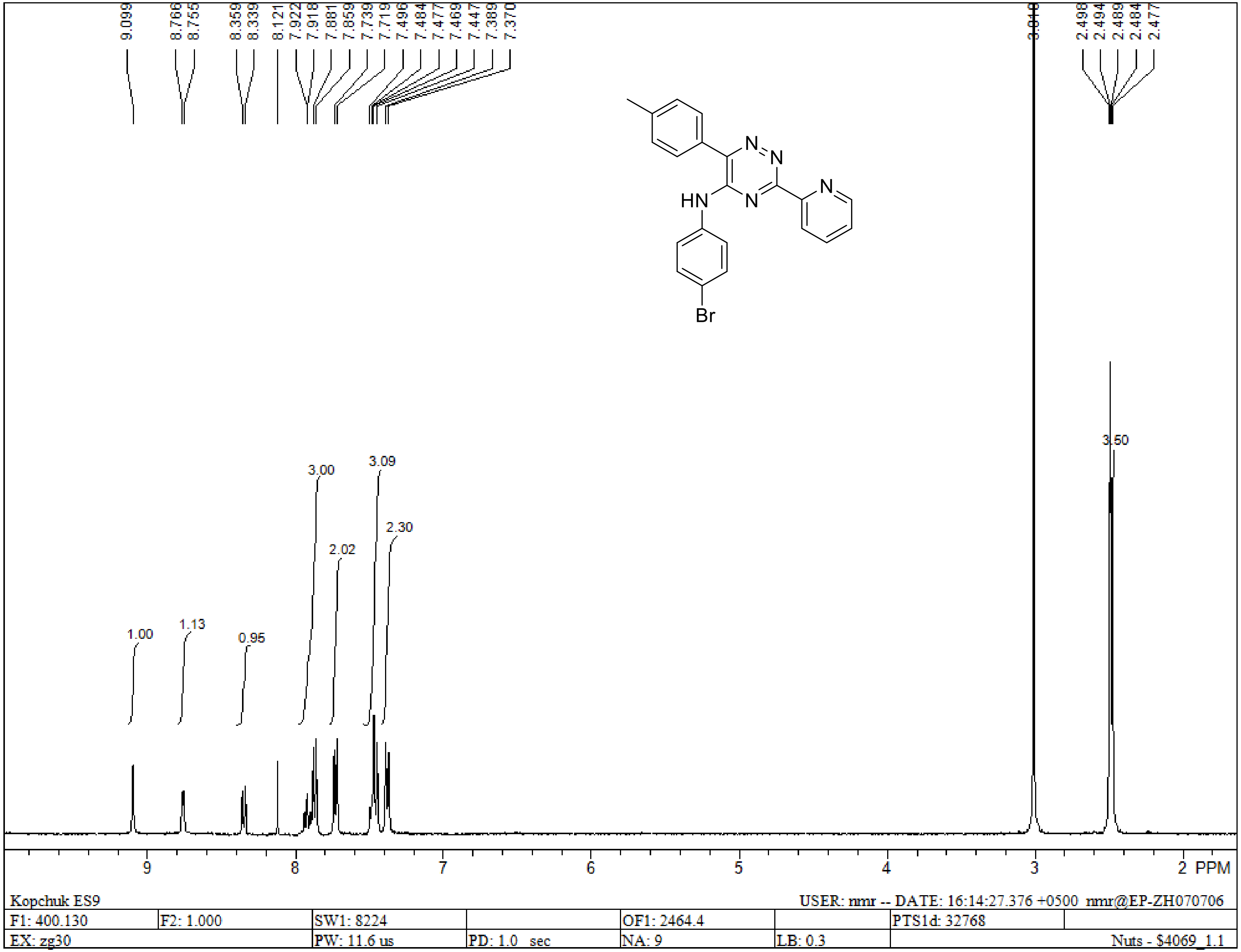

6. **Figure S3.** ^{1}H **NMR (400 MHz, DMSO-d_6) of 5c**

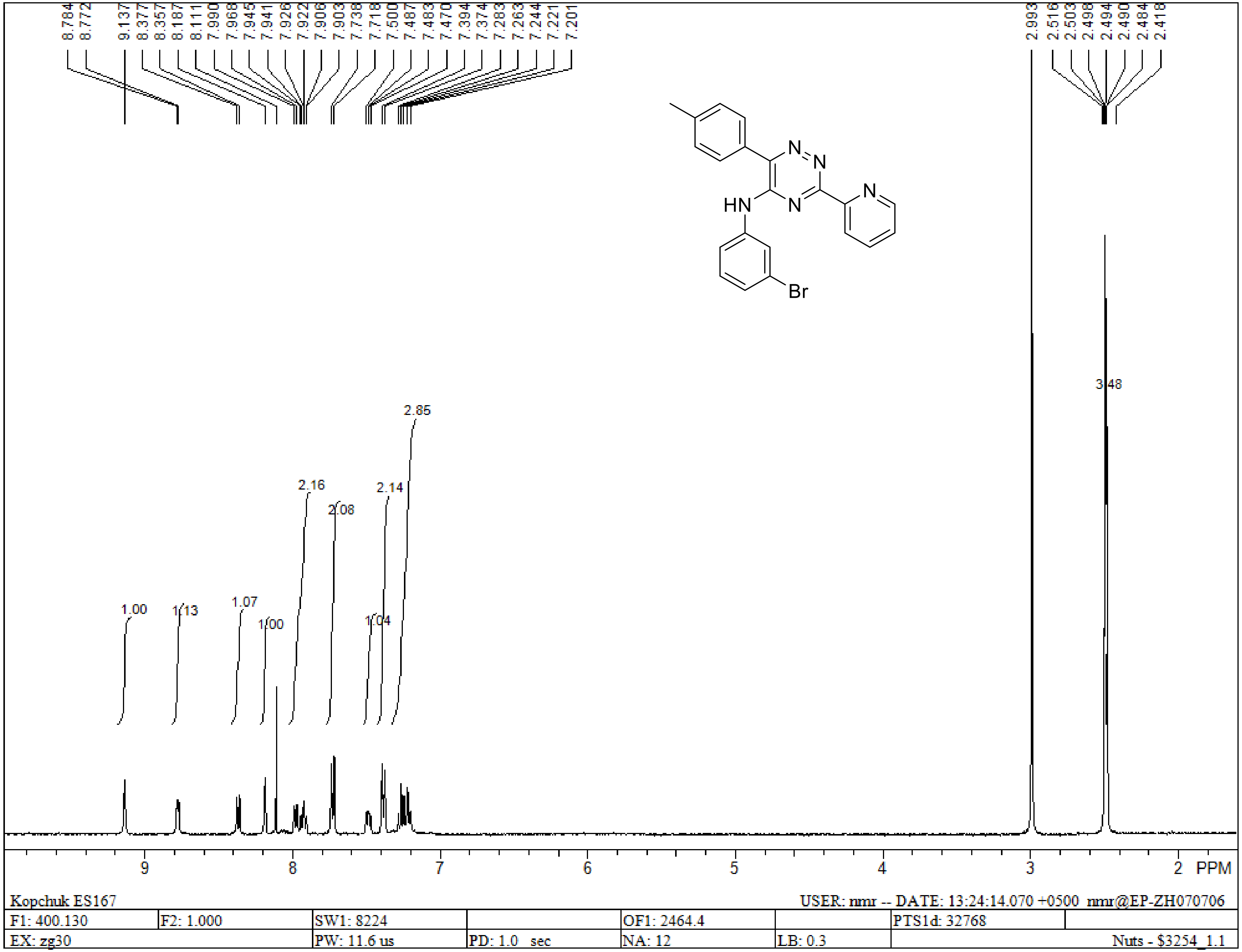

7. **Figure S4.** **^{1}H NMR (400 MHz, DMSO-d_6) of 6a**

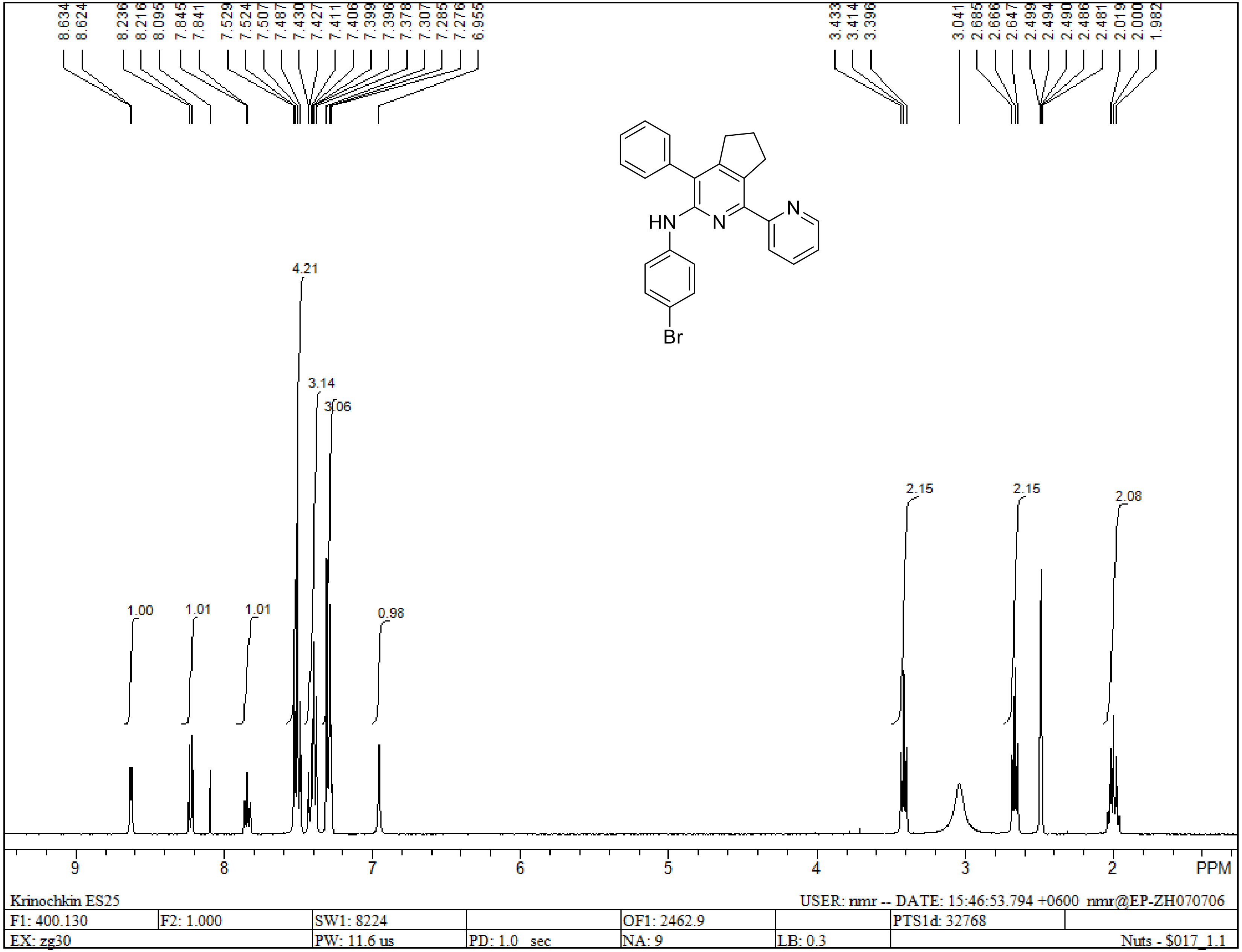

8. **Figure S5.** **^{1}H NMR (400 MHz, DMSO-d_6) of 6b**

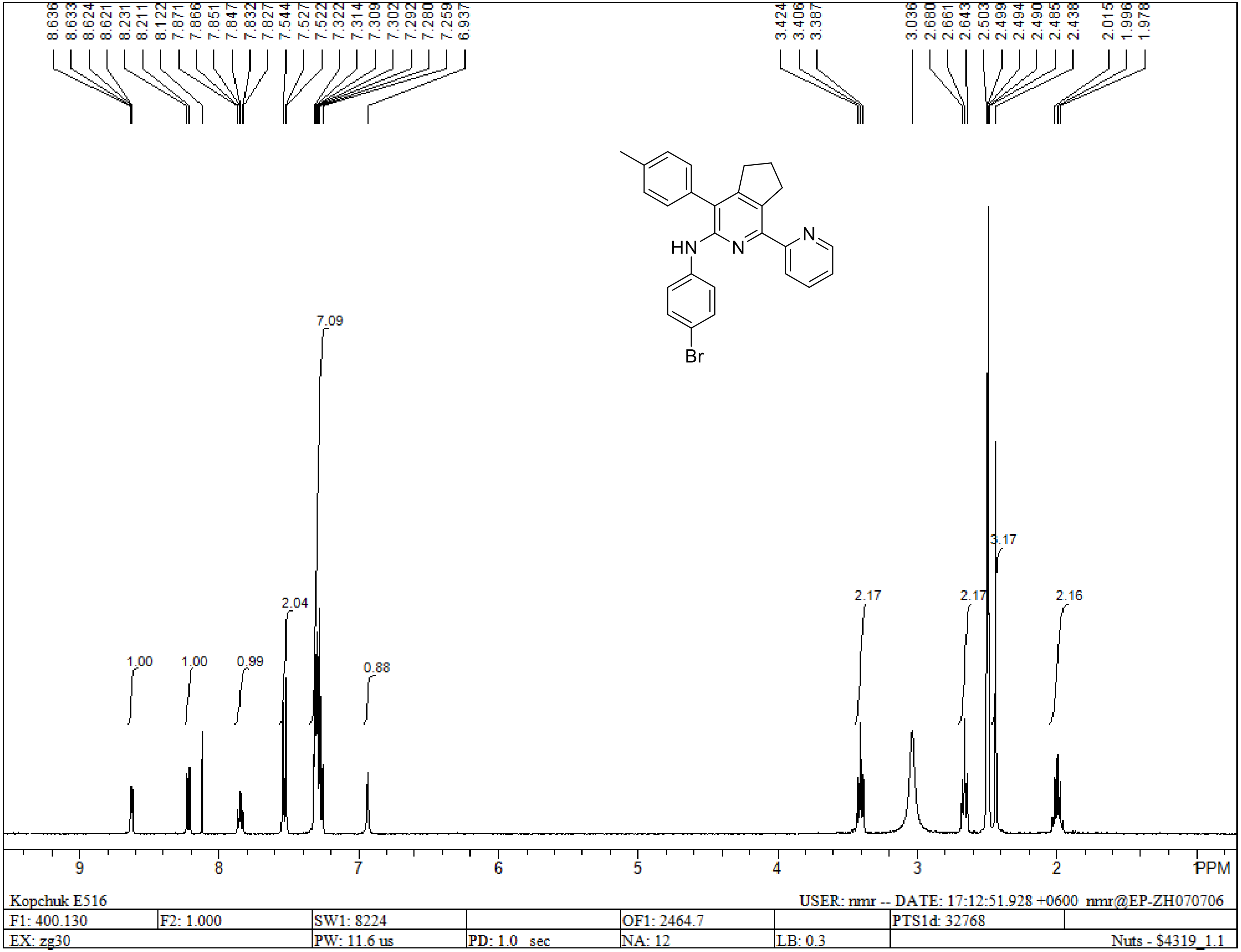

9. **Figure S6.** **^{1}H NMR (400 MHz, DMSO-d_6) of 6c**

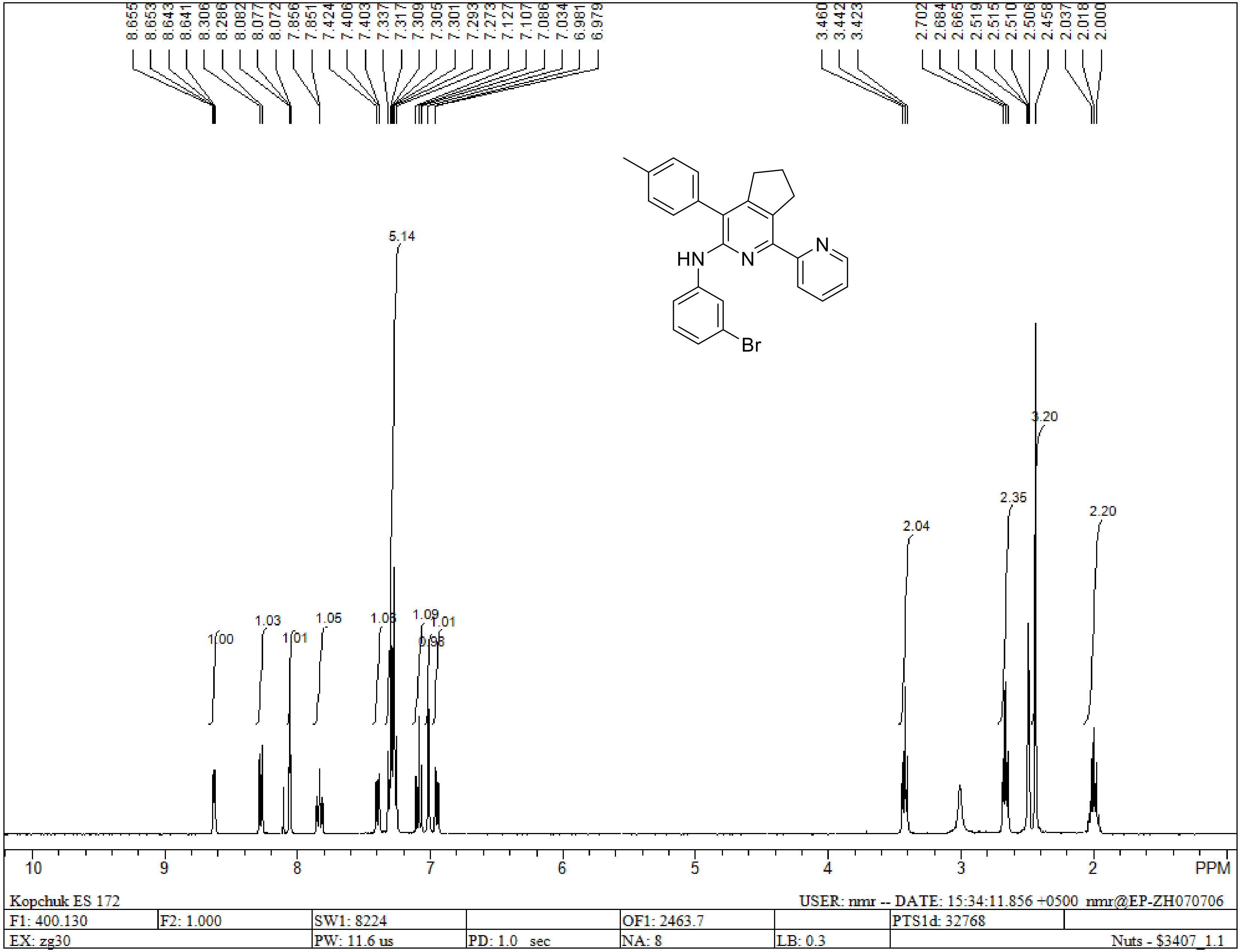

10. Figure S7. ^{1}H NMR (400 MHz, DMSO-d_6) of 3a

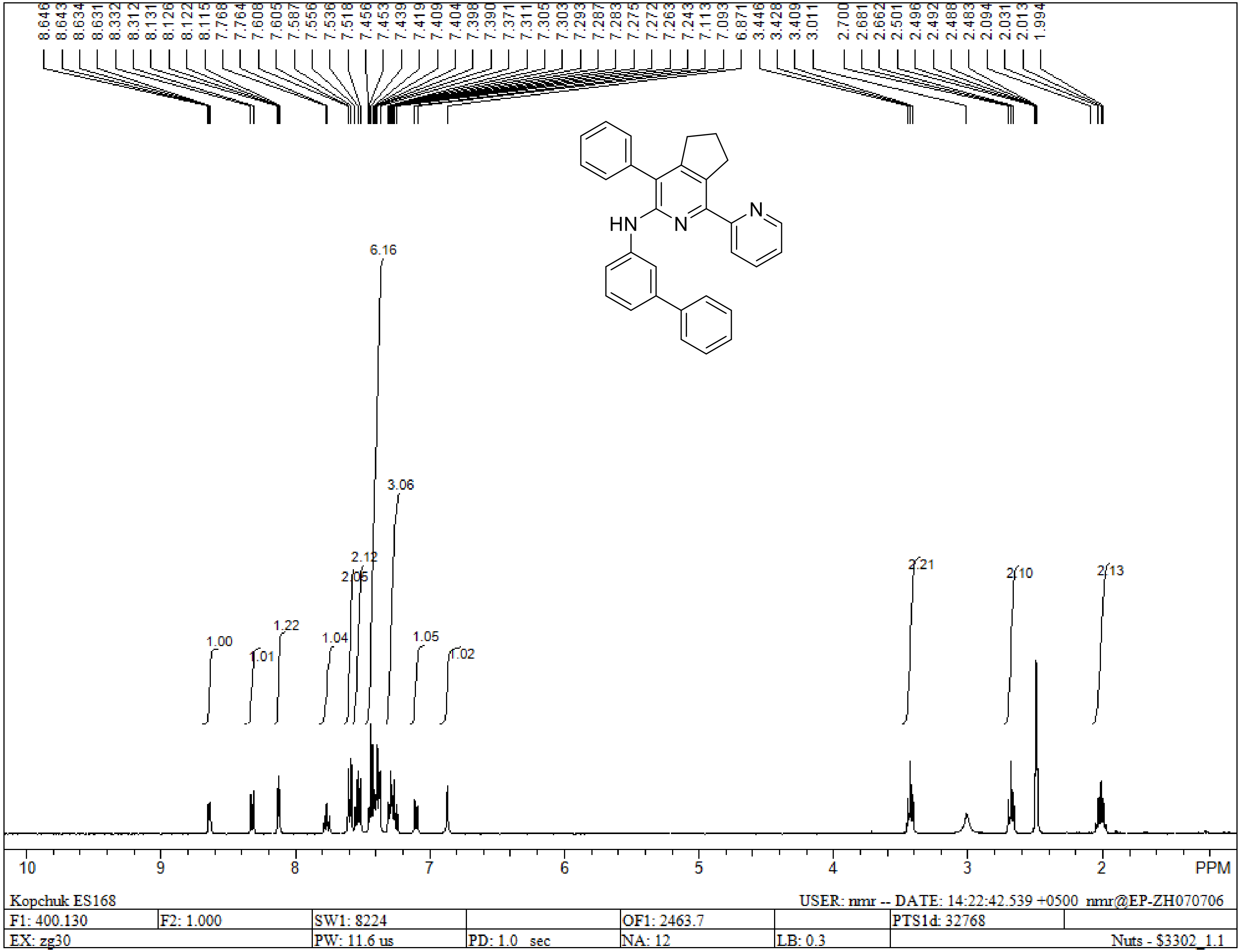

11. Figure S8. ^{13}C NMR (400 MHz, DMSO-d_6) of 3a

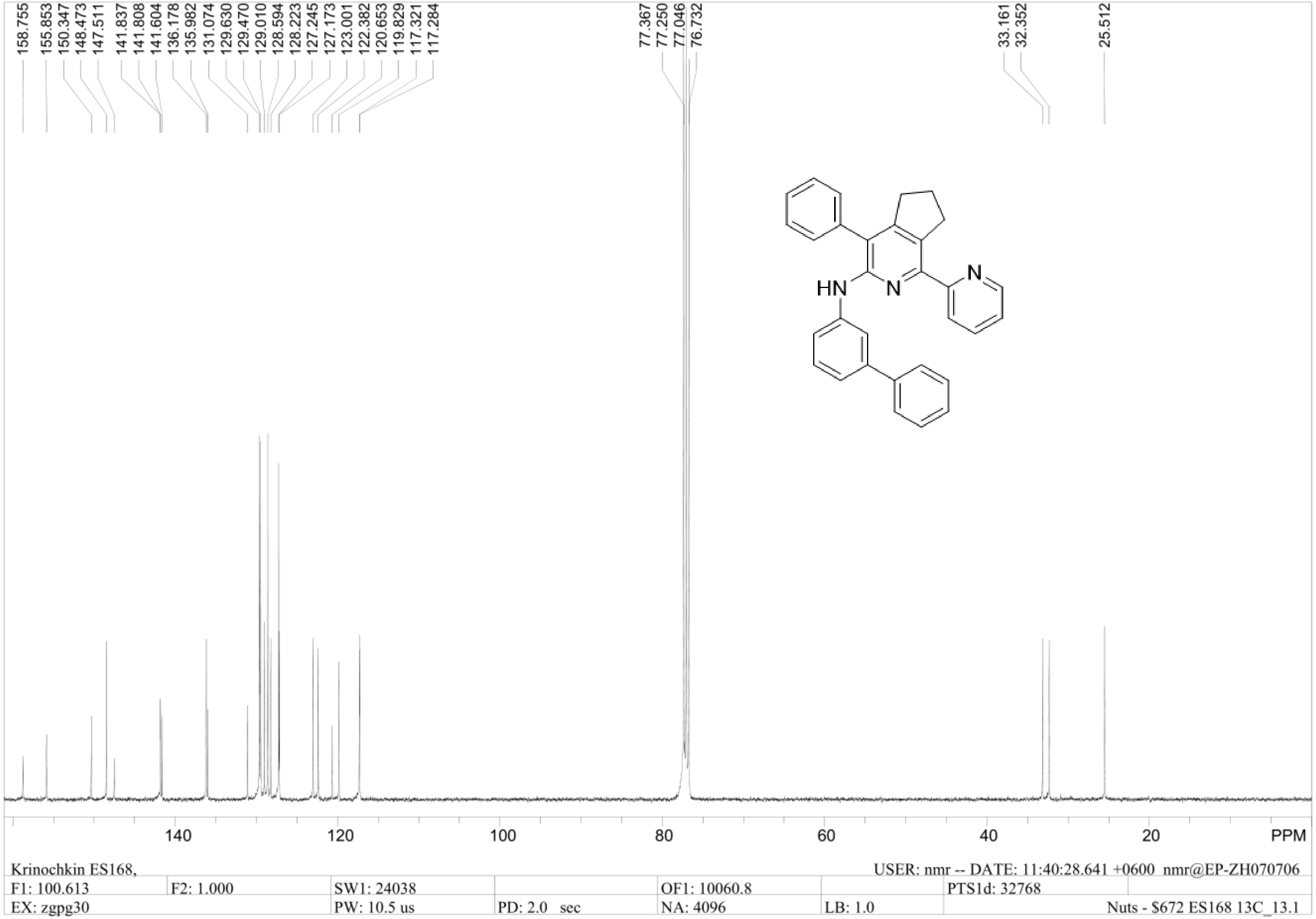

12. Figure S9.^{1}H NMR (400 MHz, DMSO-d_6) of 3b

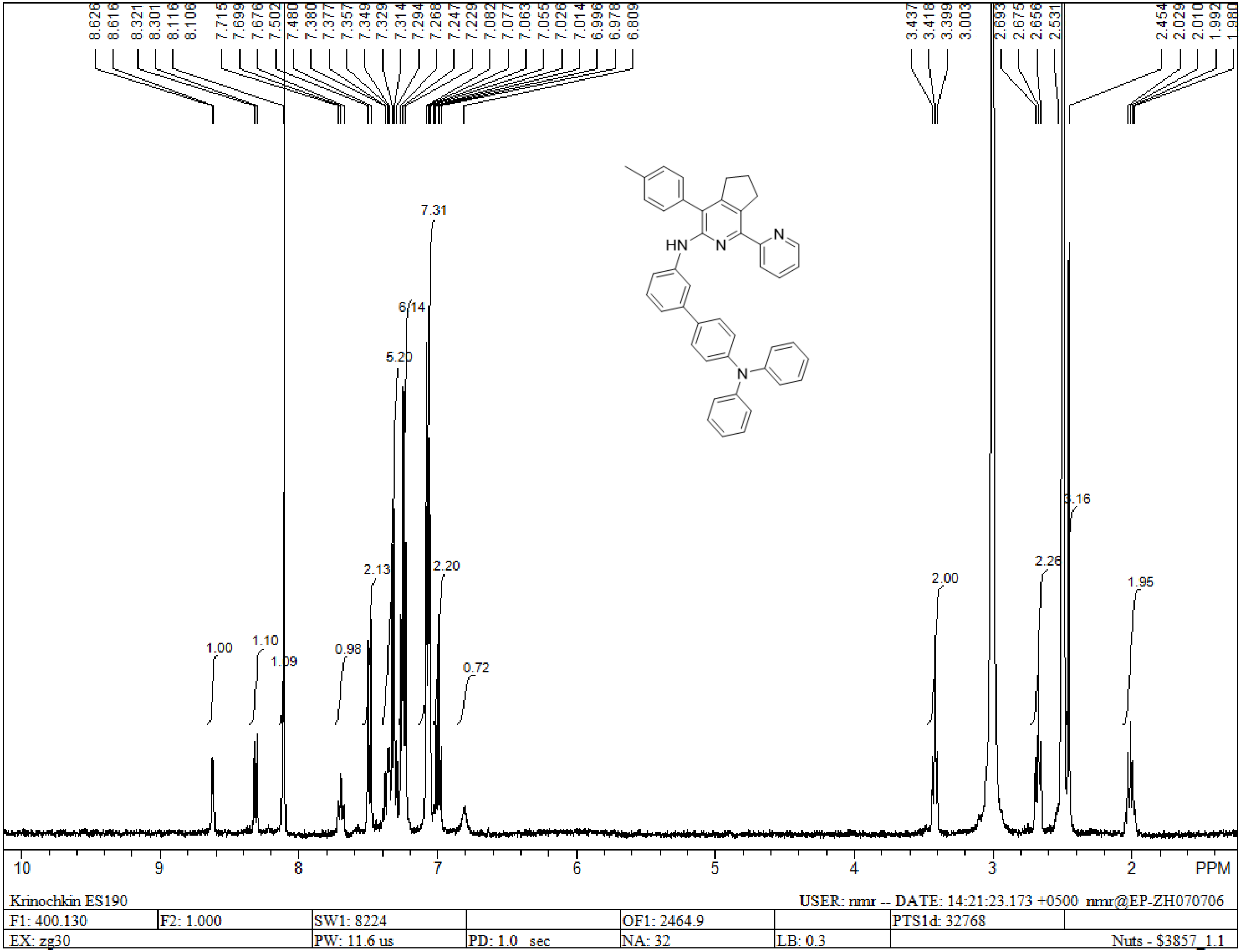

13. Figure S10.^{13}C NMR (400 MHz, DMSO-d_6) of 3b

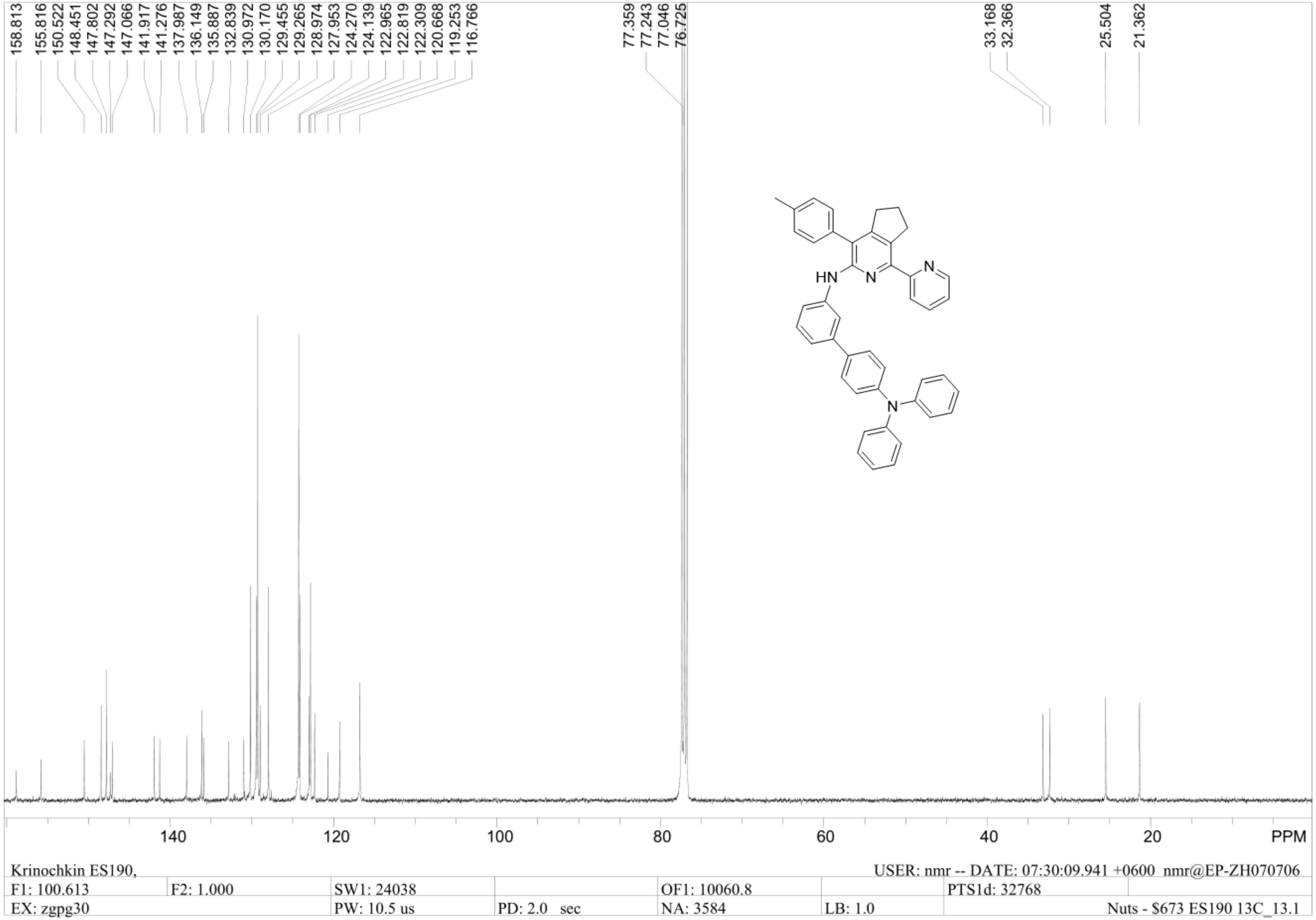

14. Figure S11. ^{1}H NMR (400 MHz, DMSO-d_6) of 3c

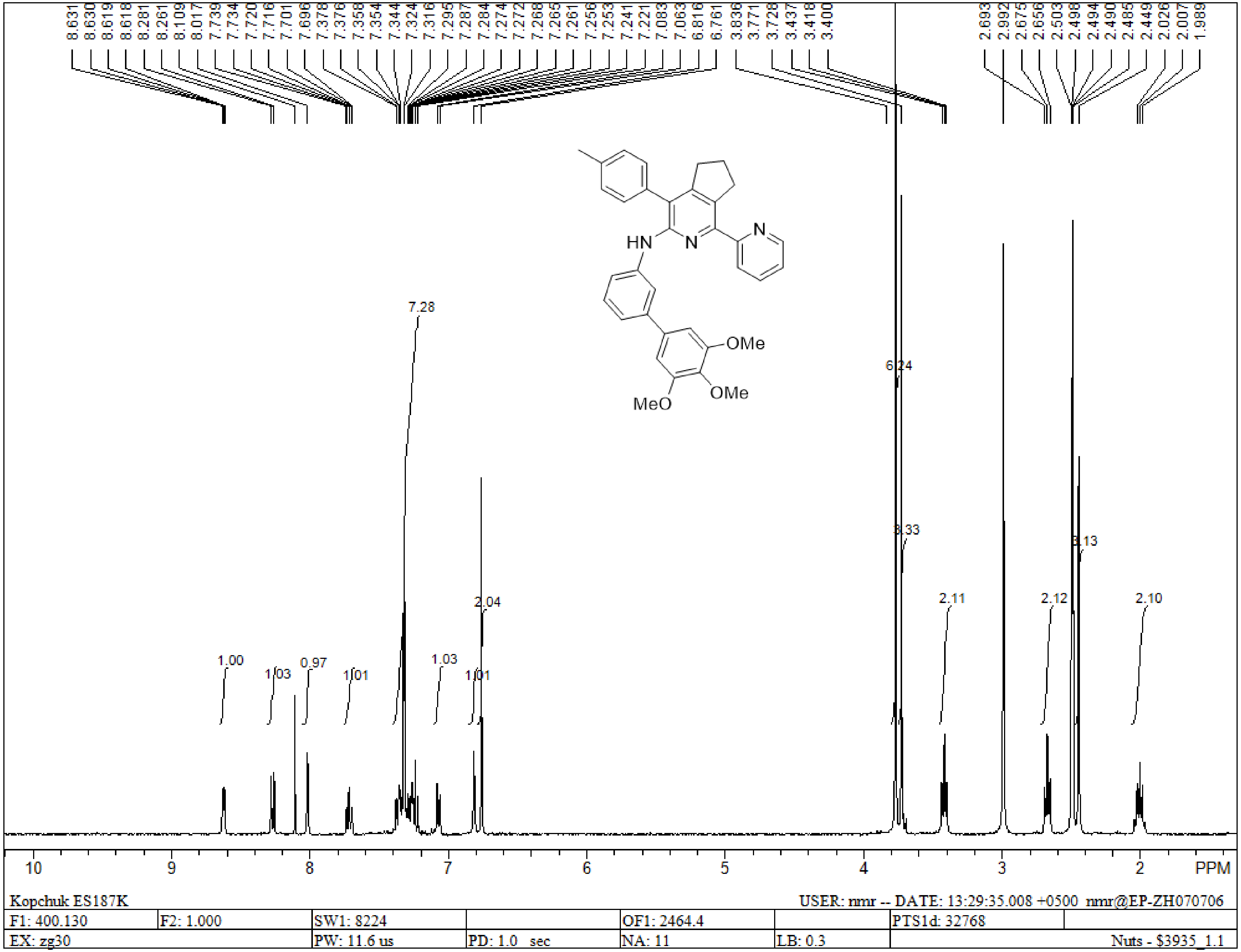

15. Figure S12.^{13}C NMR (400 MHz, DMSO-d_6) of 3c

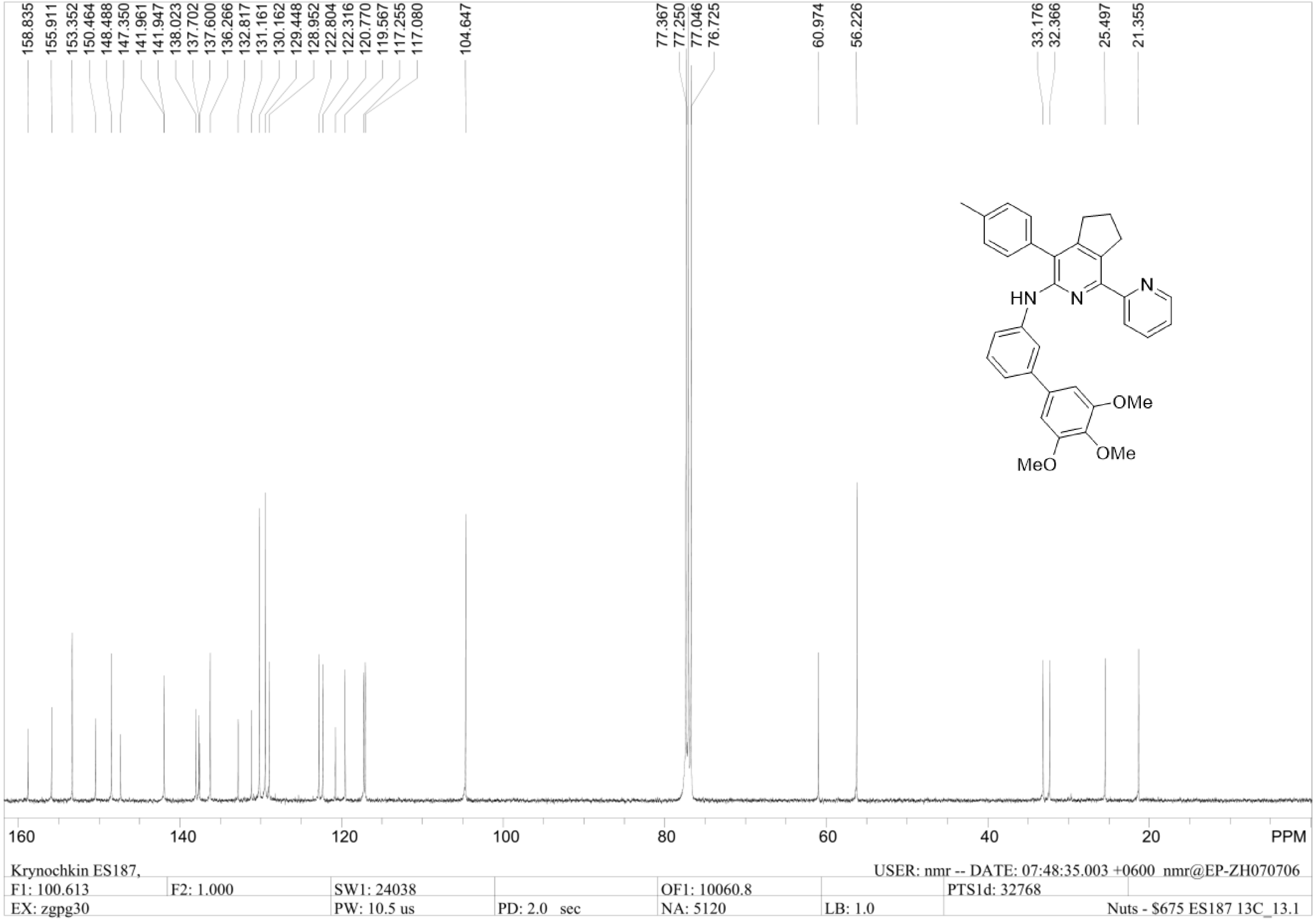

16. Figure S13. ^{1}H NMR (400 MHz, DMSO-d_6) of 3d

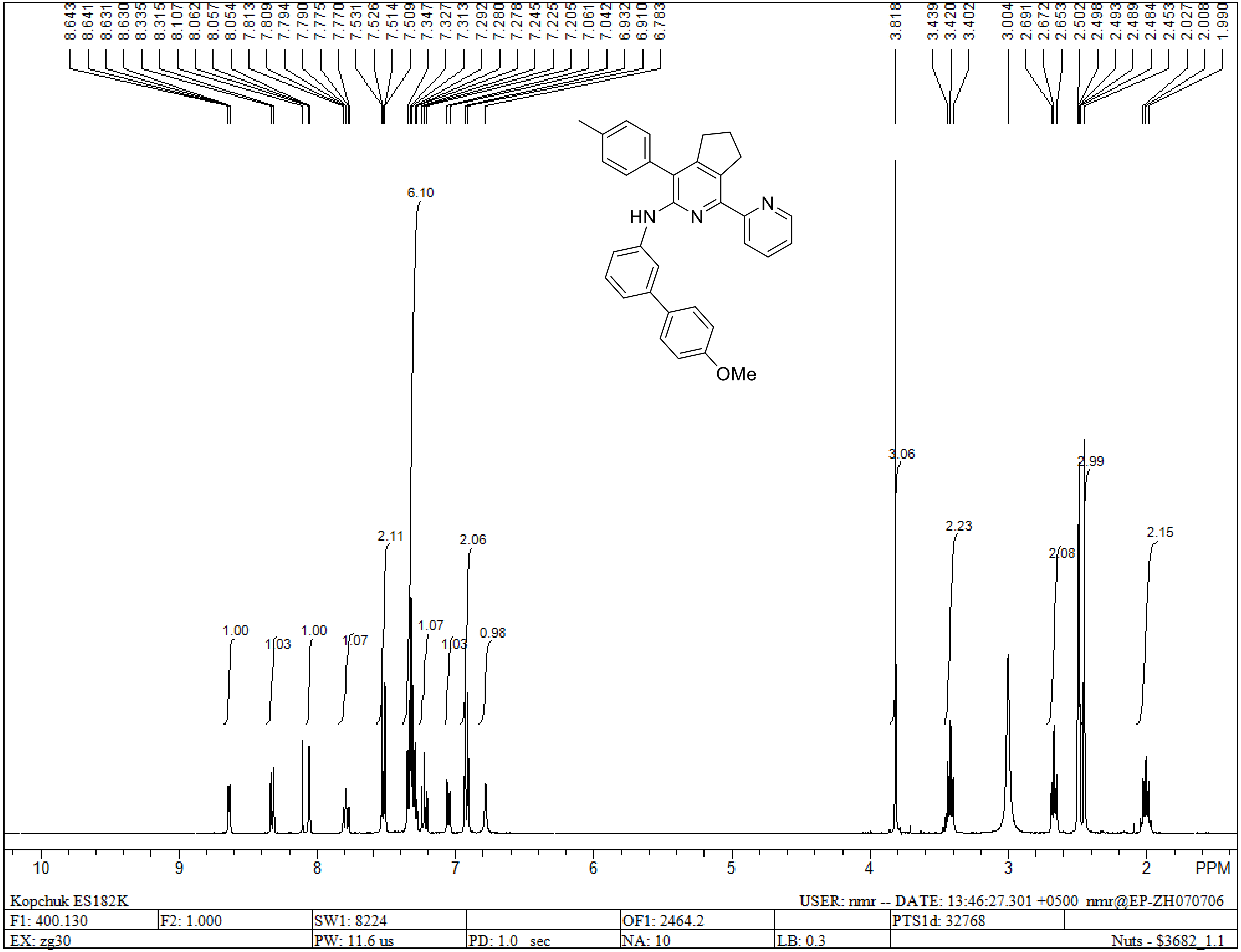

17. Figure S14.^{13}C NMR (400 MHz, DMSO-d_6) of 3d

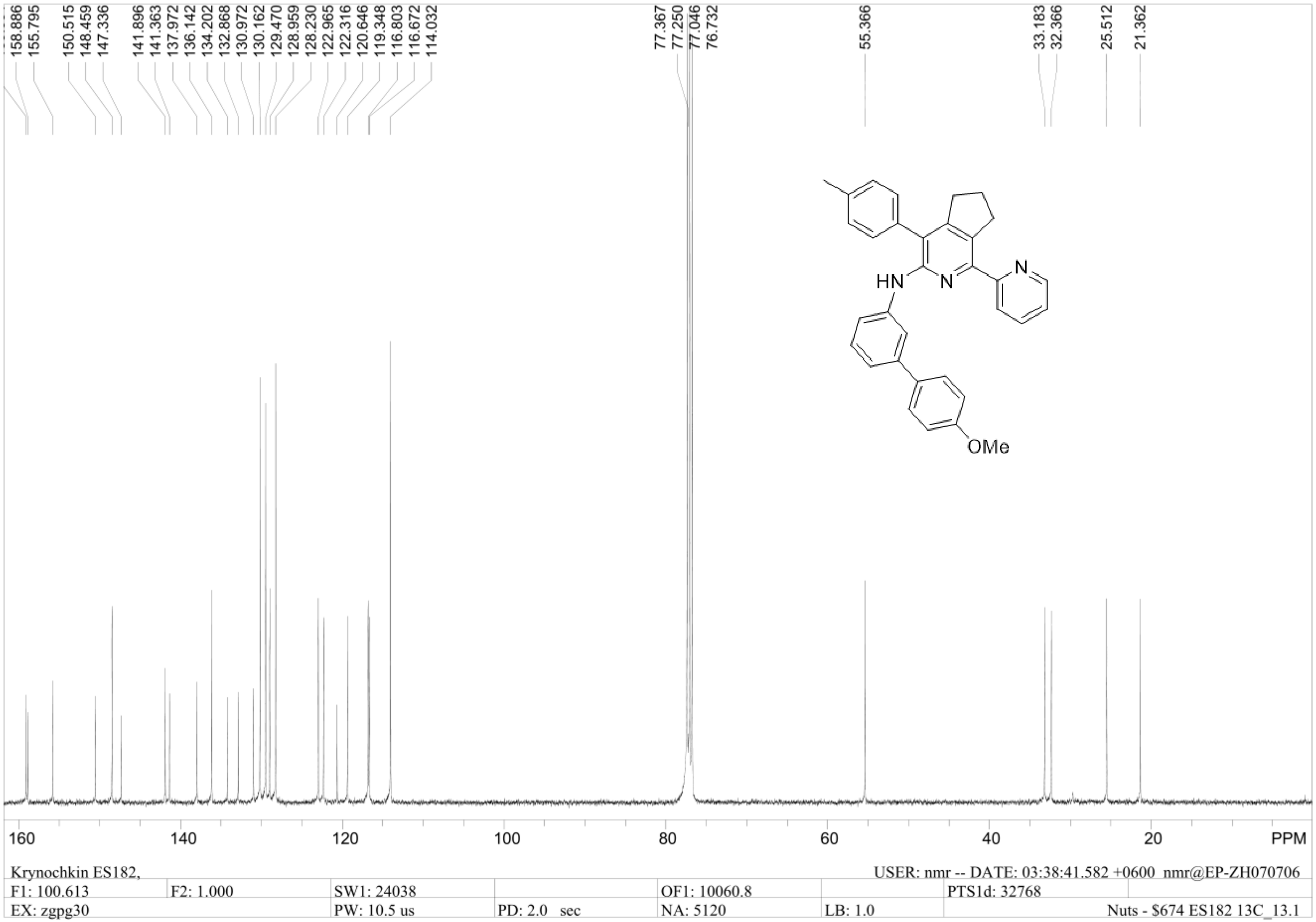

18. Figure S15.^{1}H NMR (400 MHz, DMSO-d_6) of 3e

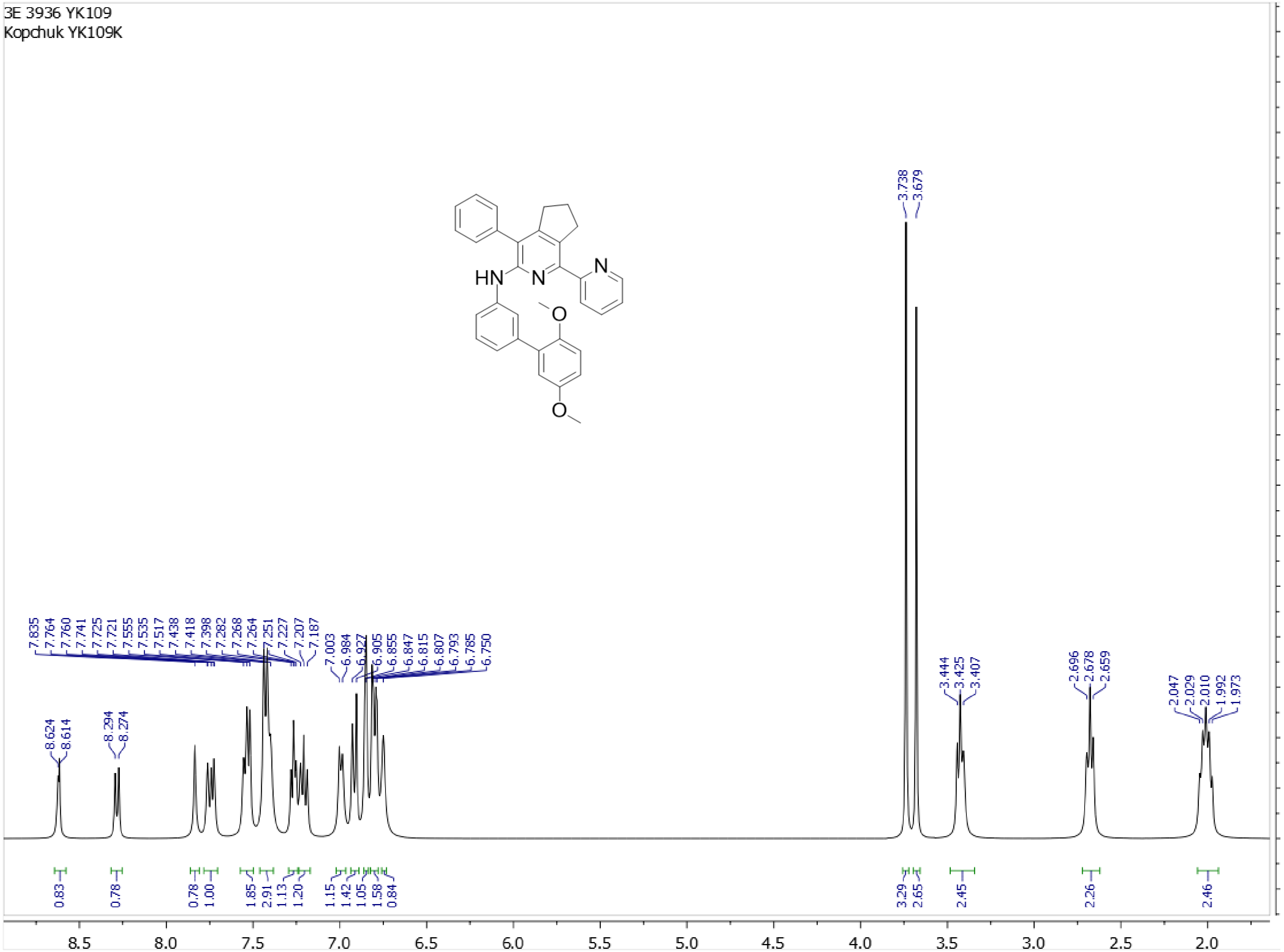

19. Figure S16.^{13}C NMR (400 MHz, DMSO-d_6) of 3e

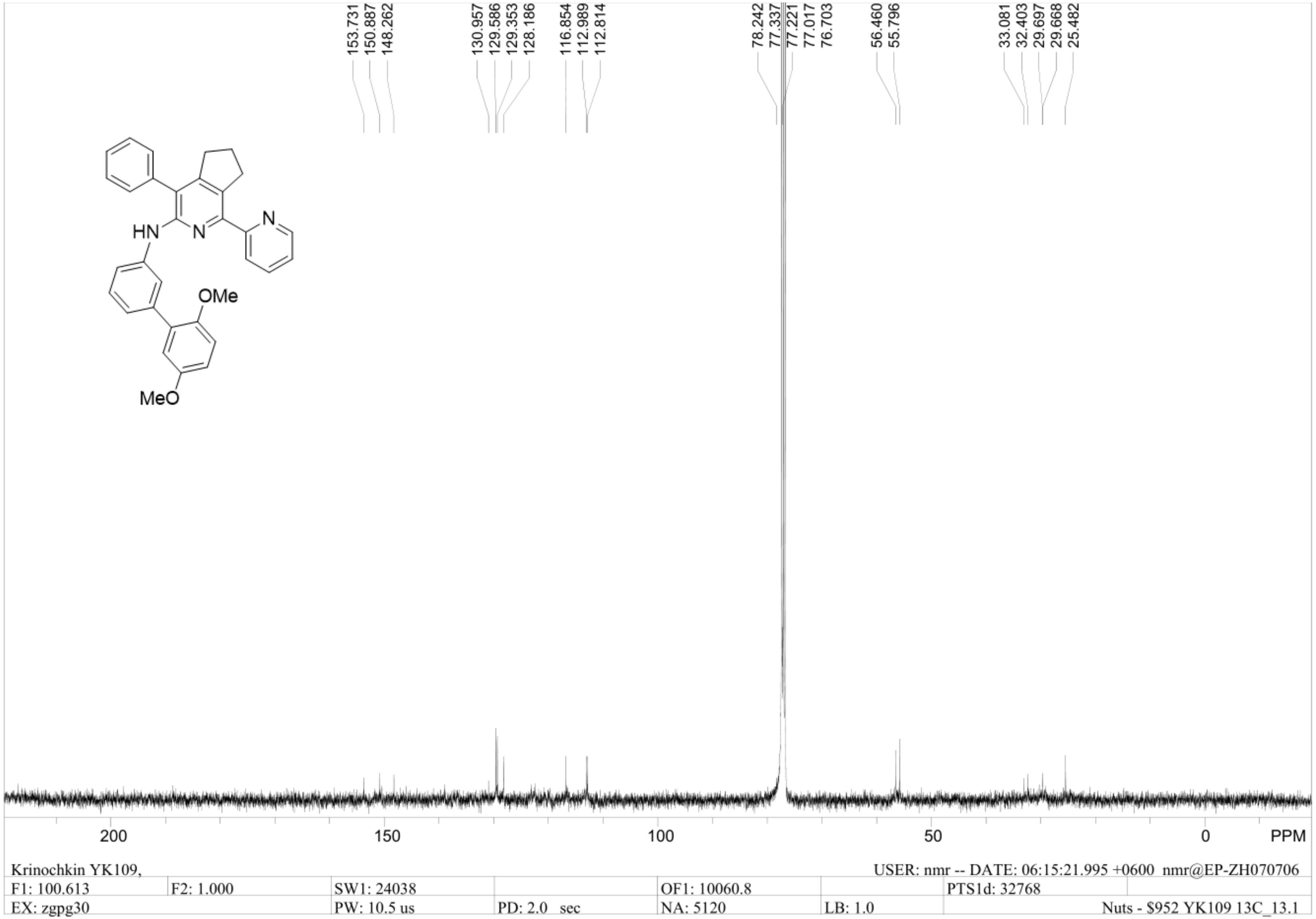

20. Figure S17.^{1}H NMR (400 MHz, DMSO-d_6) of 3f

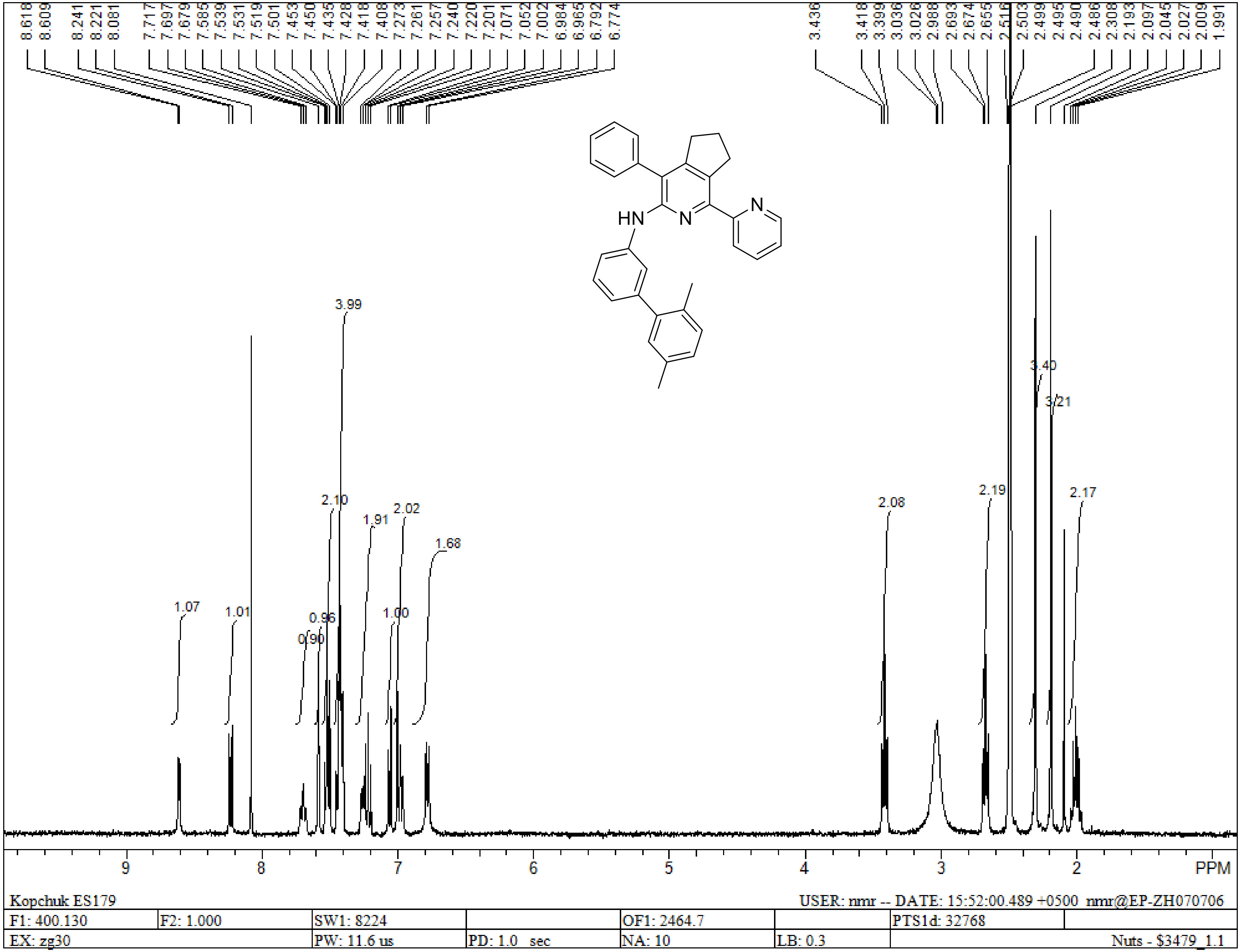

21. Figure S18.^{13}C NMR (400 MHz, DMSO-d_6) of 3f

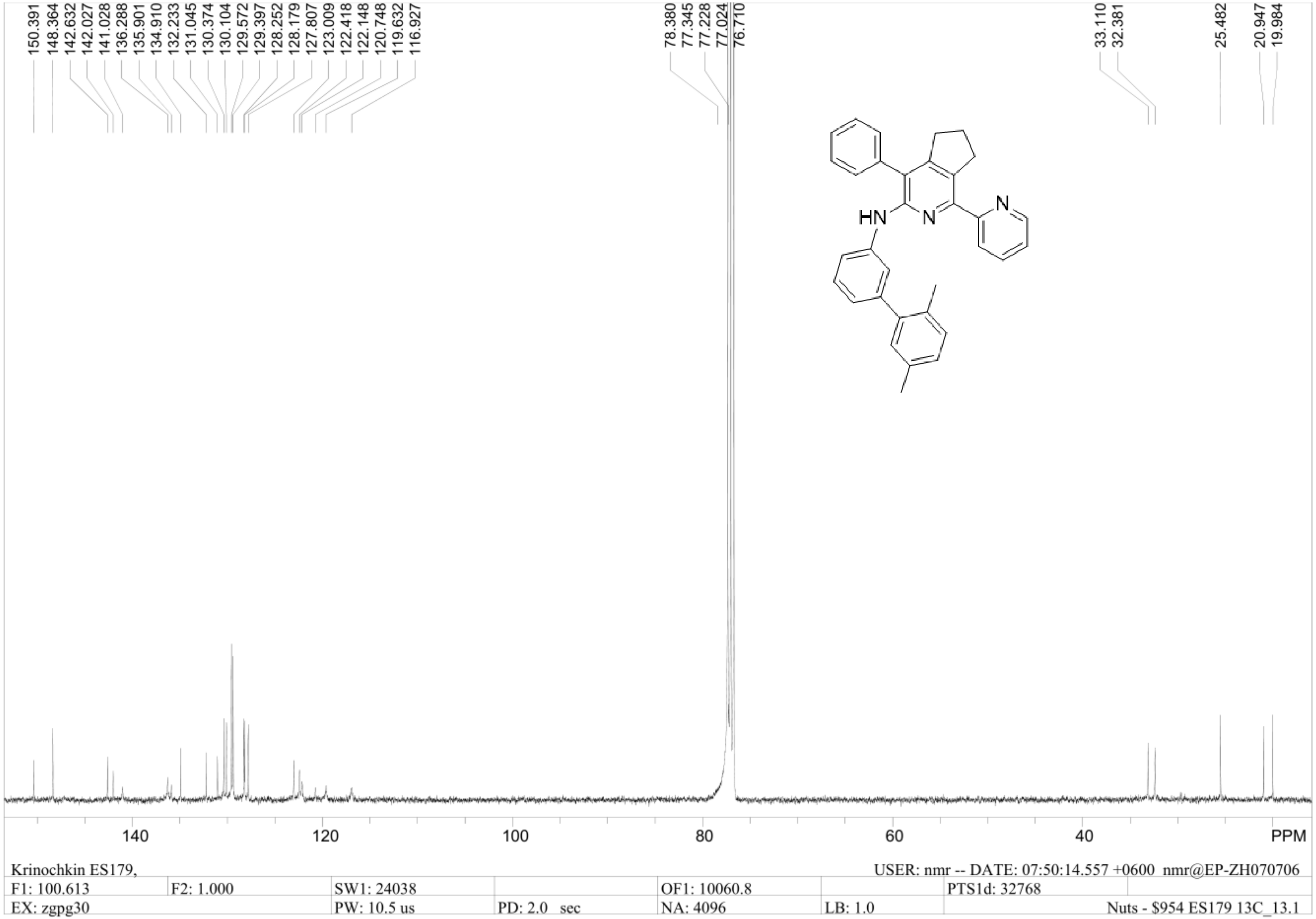

22. **Figure S19.^{1}H NMR (400 MHz, DMSO-d_6) of 3g**

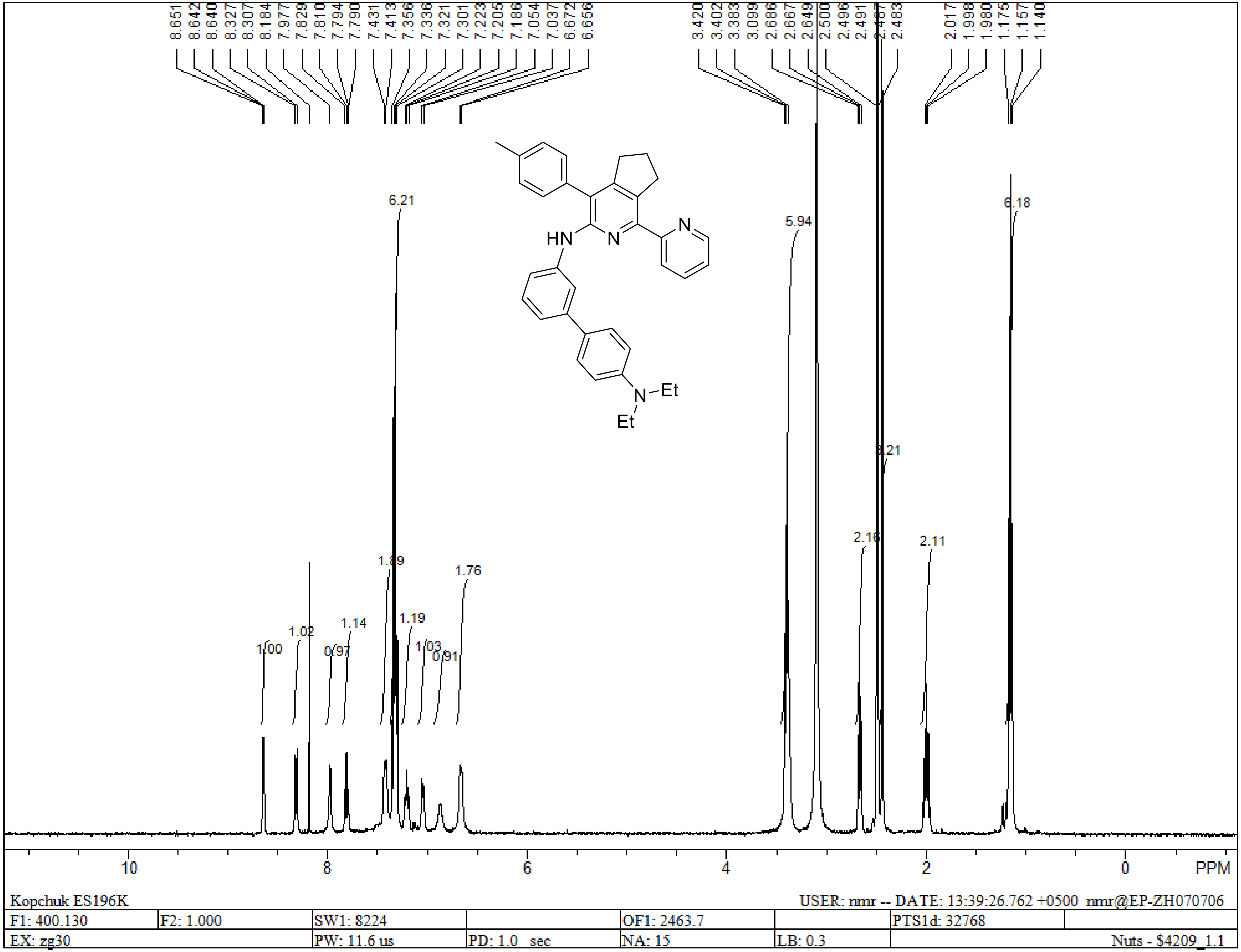

23. Figure S20. ^{13}C NMR (400 MHz, DMSO-d_6) of 3g

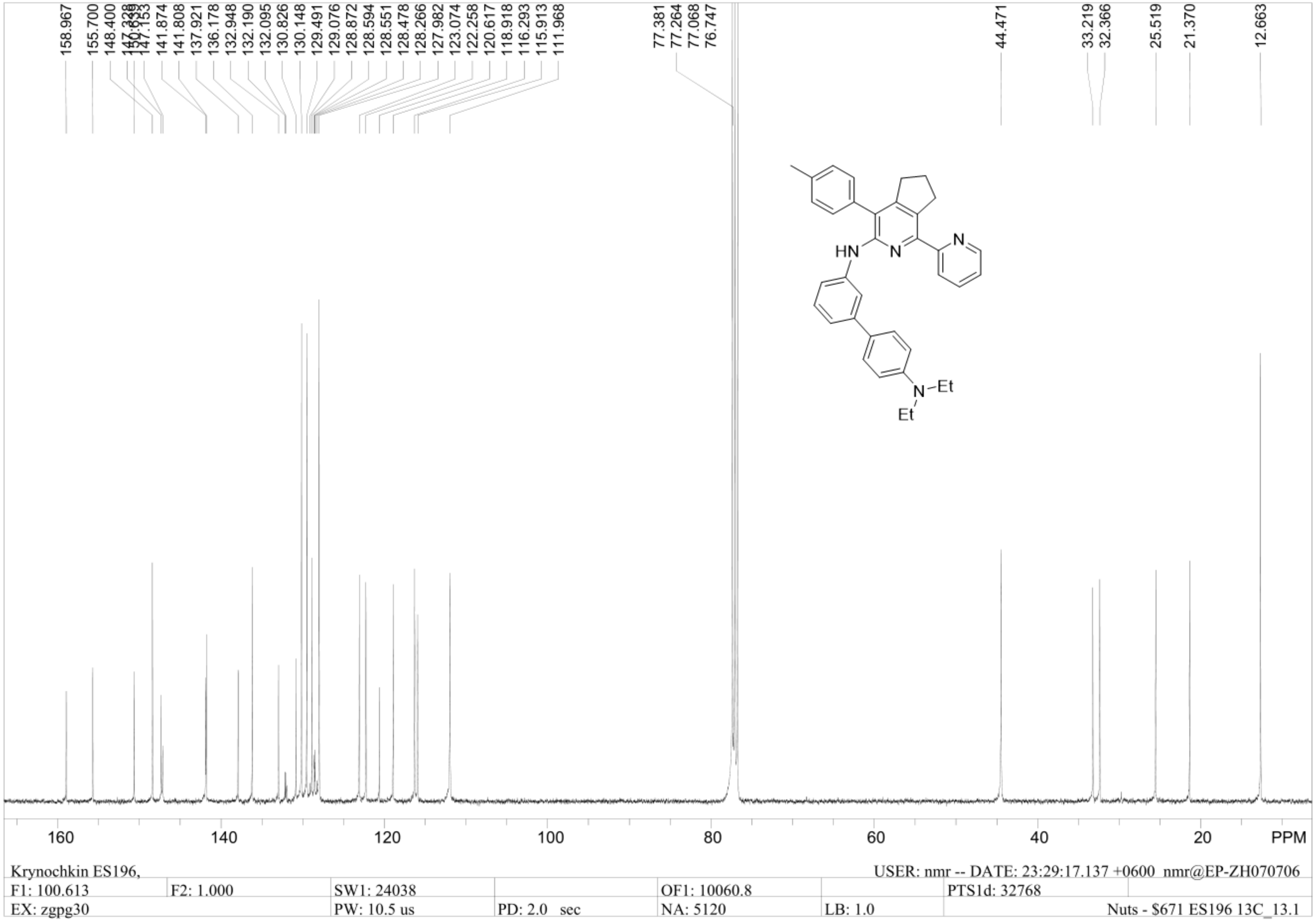

24. Figure S21.^{1}H NMR (400 MHz, DMSO-d_6) of 3h

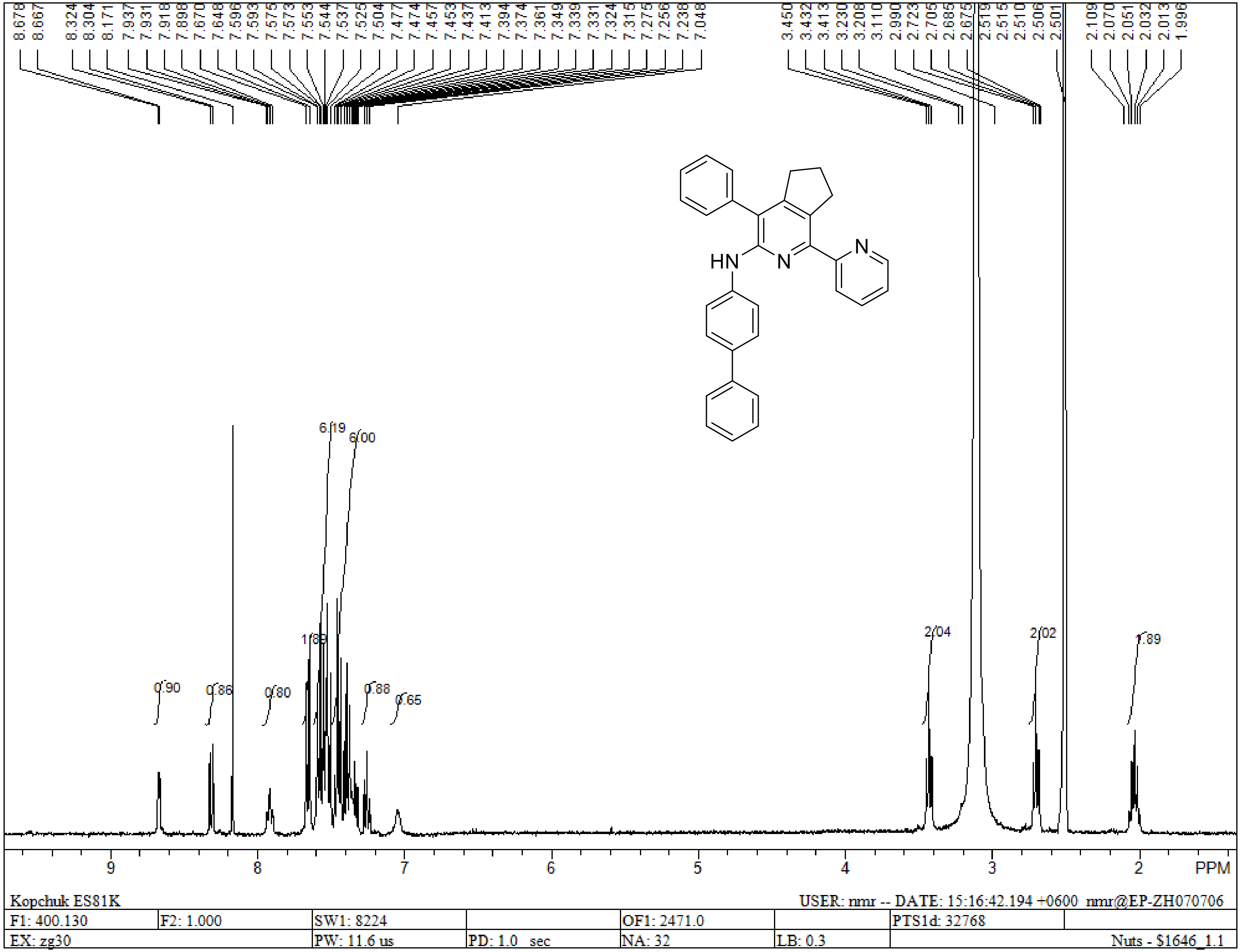

25. Figure S22.^{13}C NMR (400 MHz, DMSO-d_6) of 3h

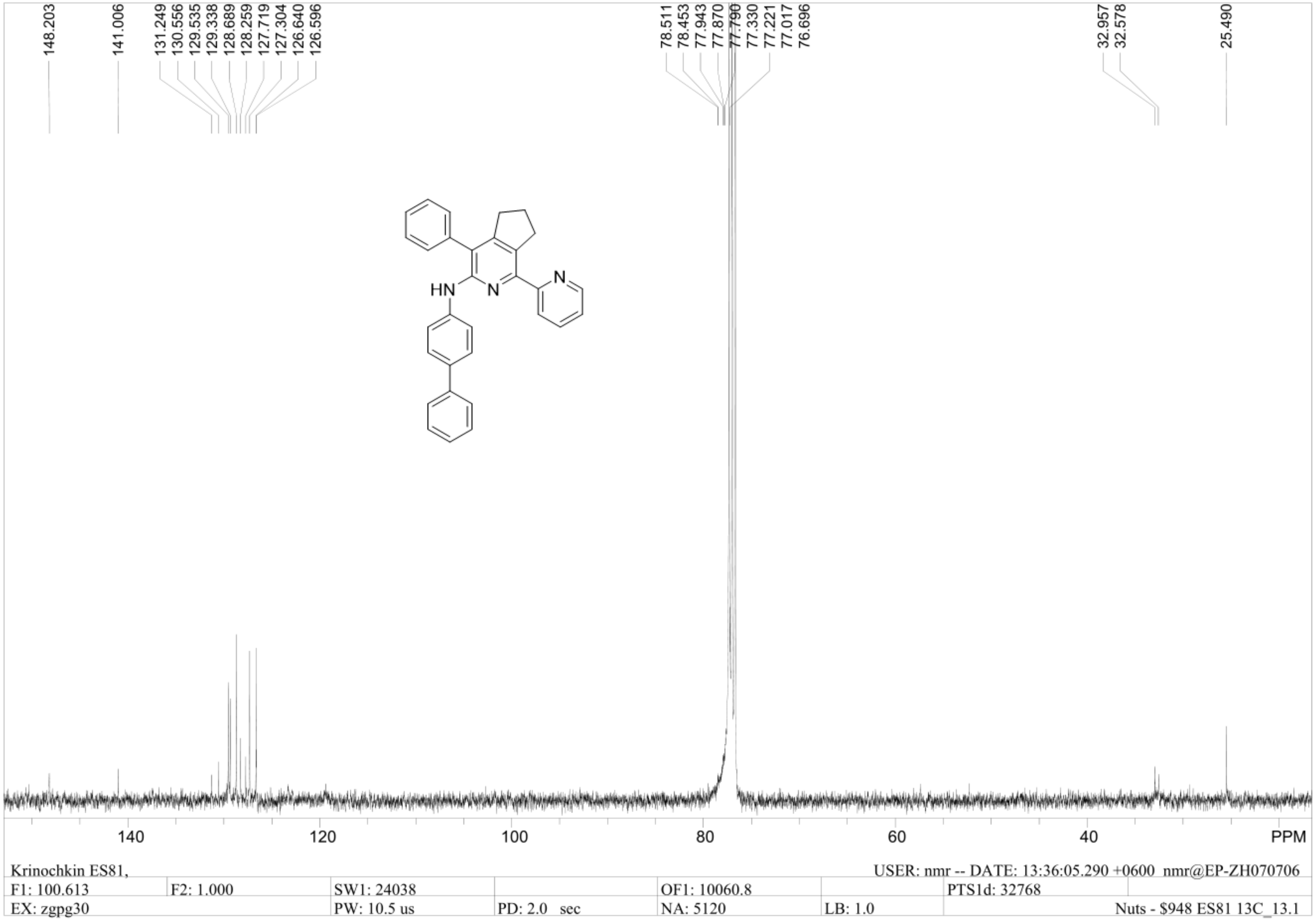

26. Figure S23.^{1}H NMR (400 MHz, DMSO-d_6) of 3i

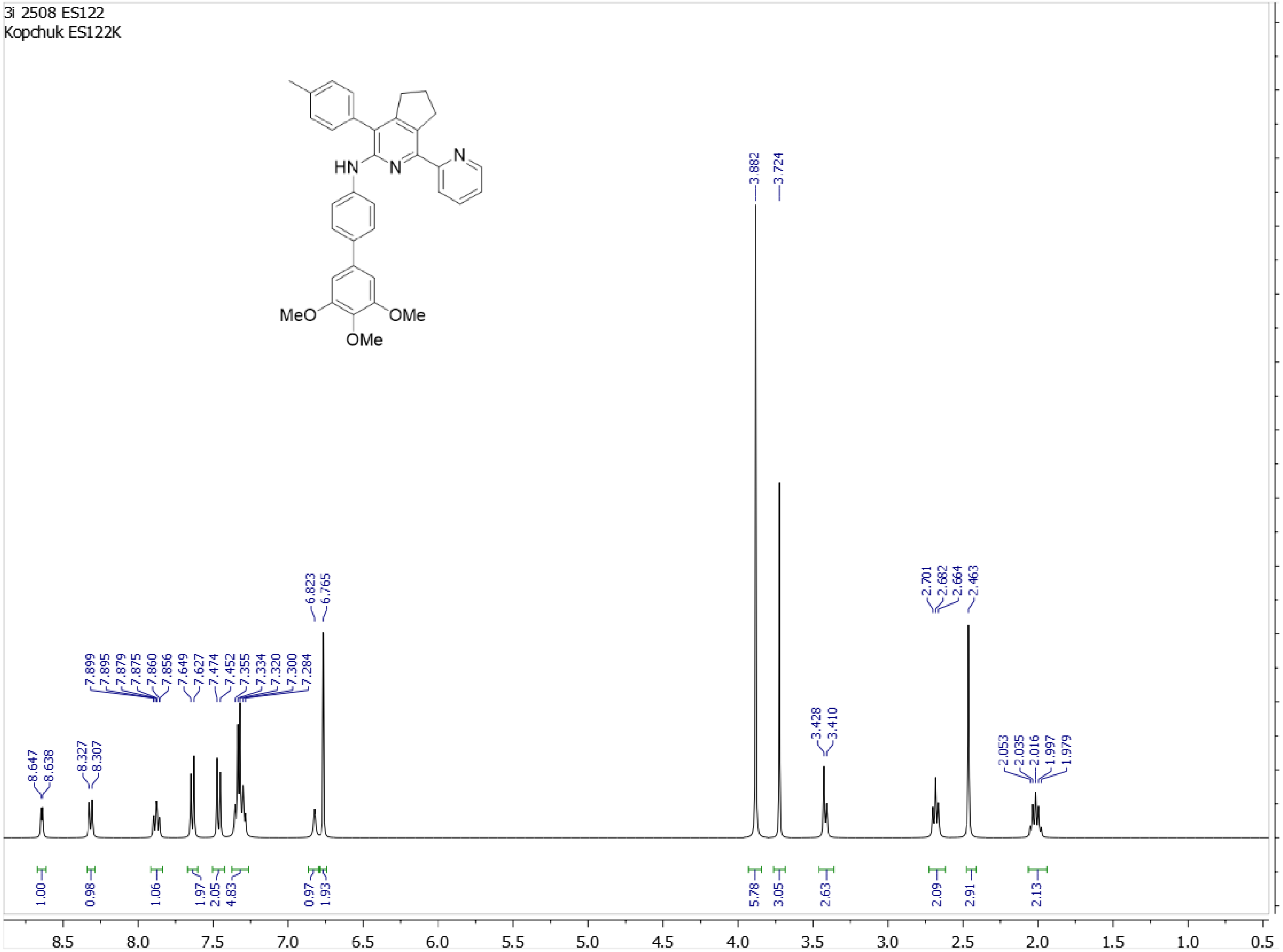

27. **Figure S24.**[13]**C NMR (400 MHz, DMSO-d_6) of 3i**

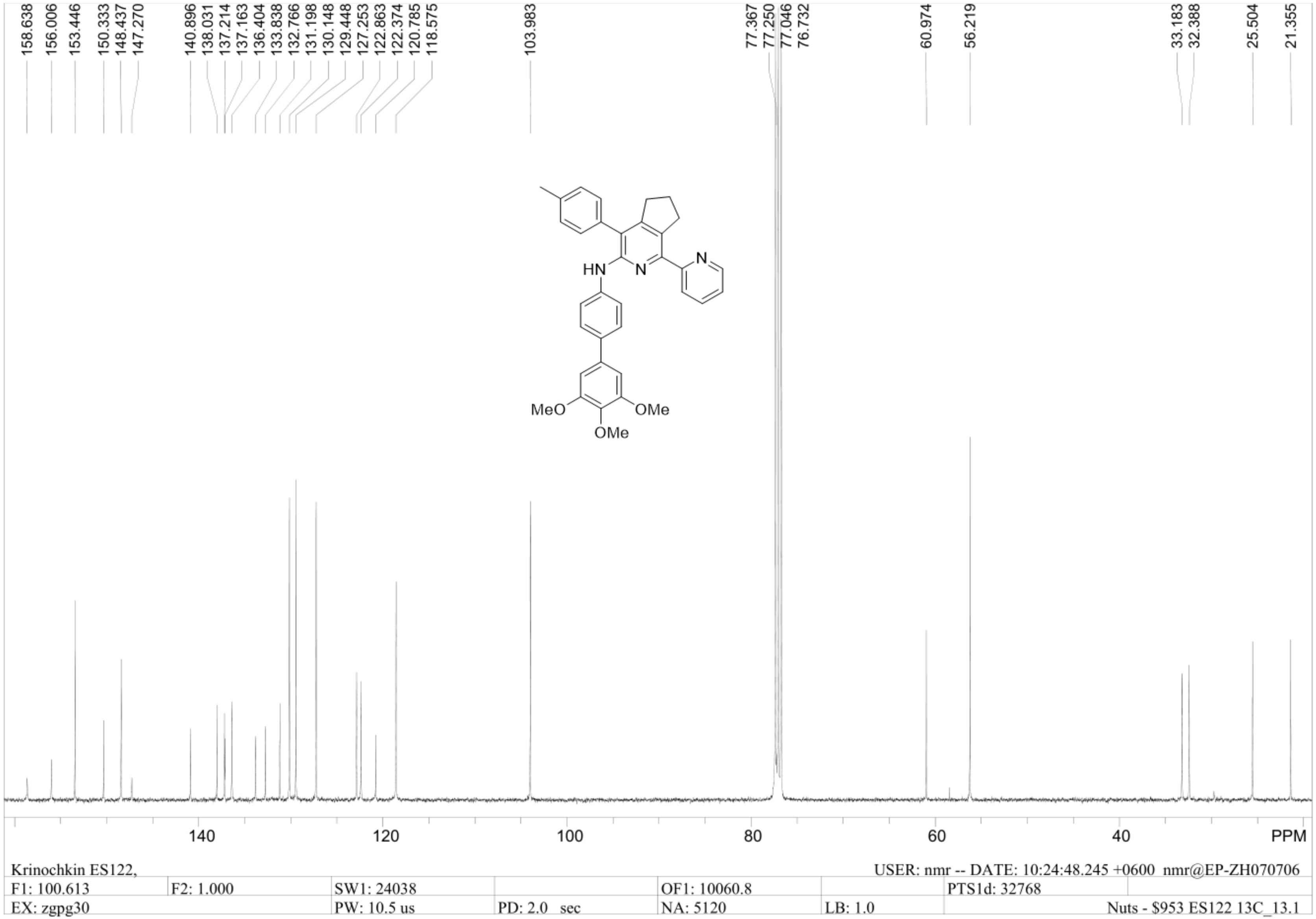

28. Figure S25.^{1}H NMR (400 MHz, DMSO-d_6) of 3j

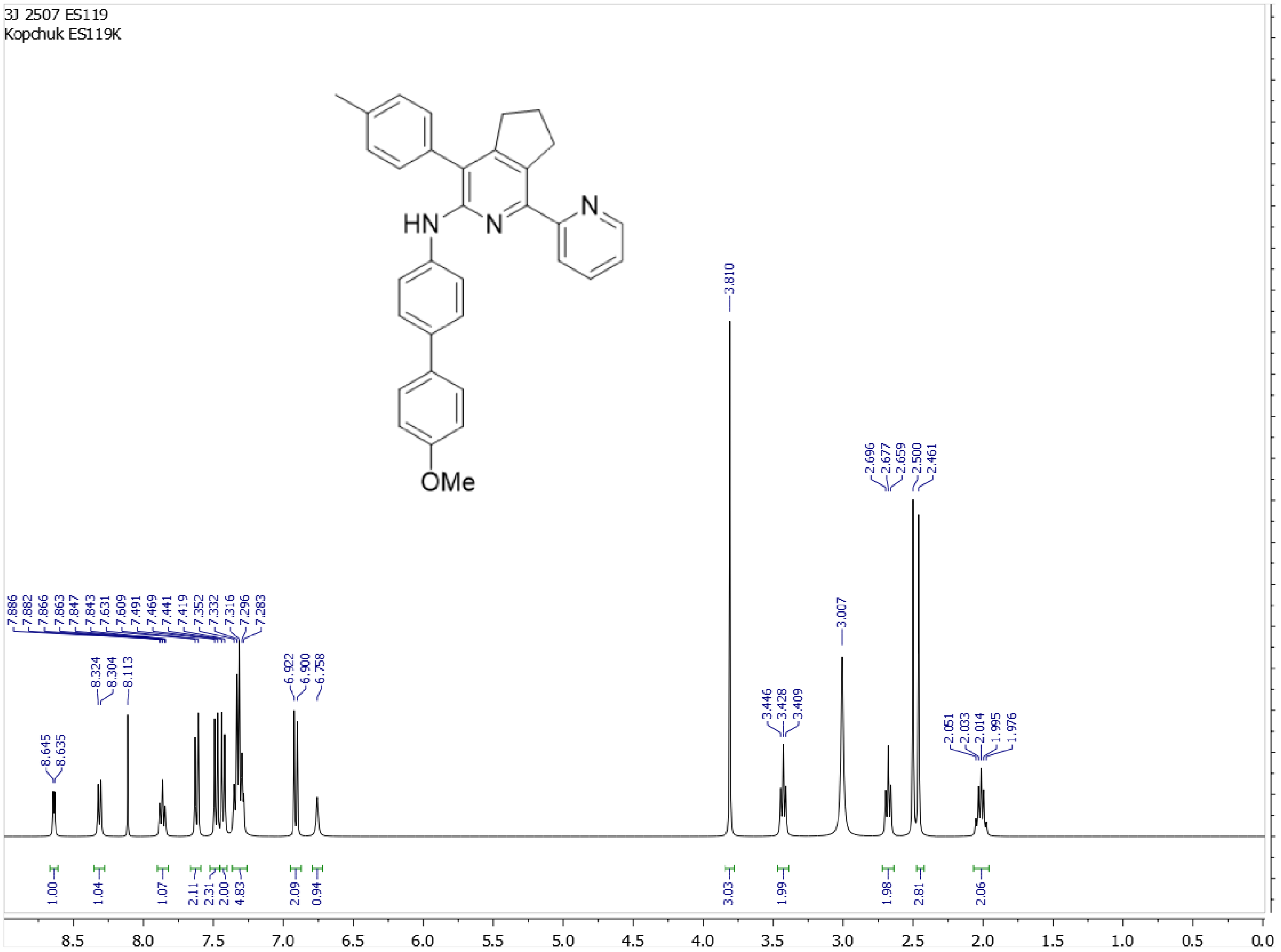

29. Figure S26.[13]**C NMR (400 MHz, DMSO-*d*$_6$) of 3j**

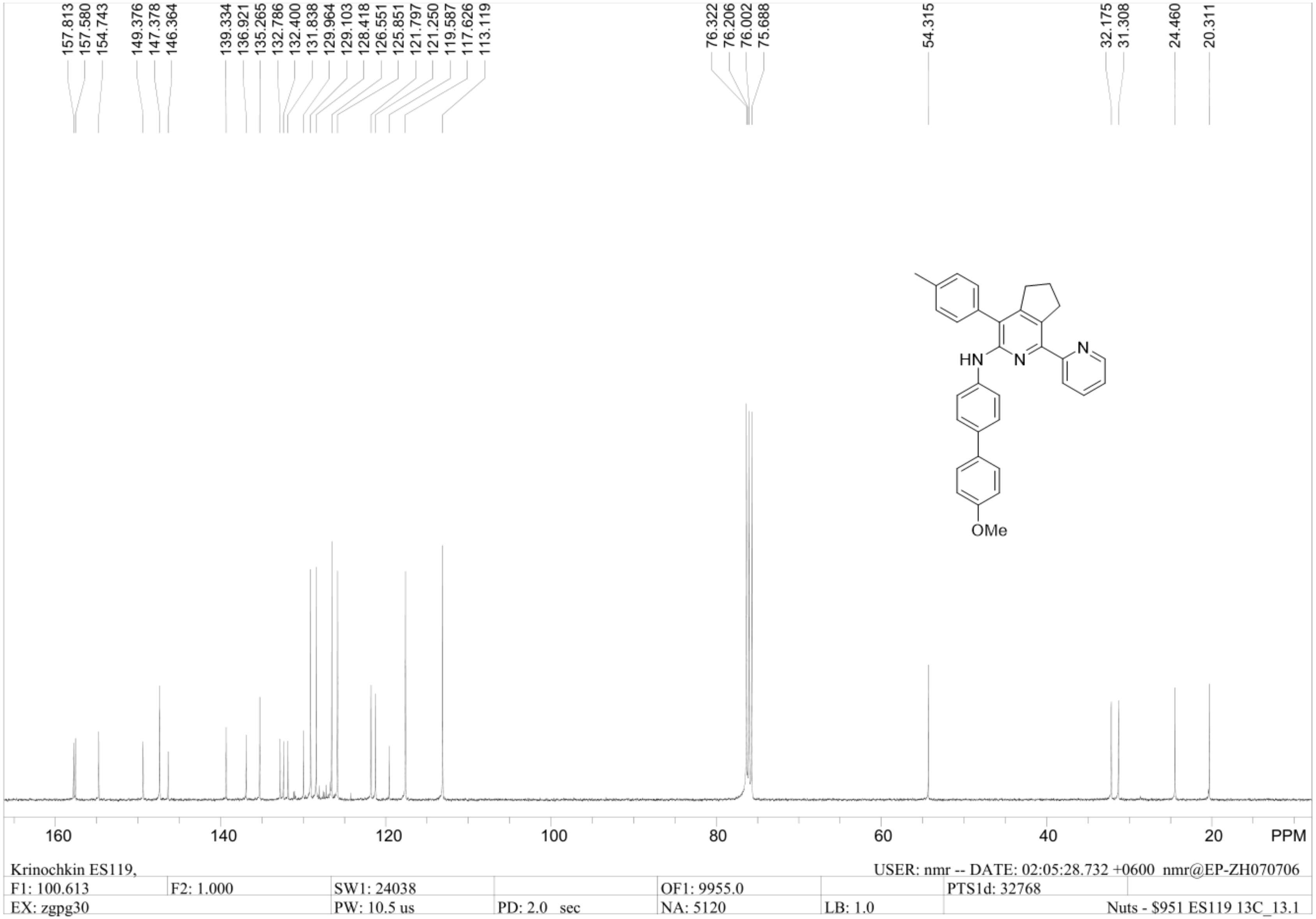

30. Figure S27.^{1}H NMR (400 MHz, DMSO-d_6) of 3k

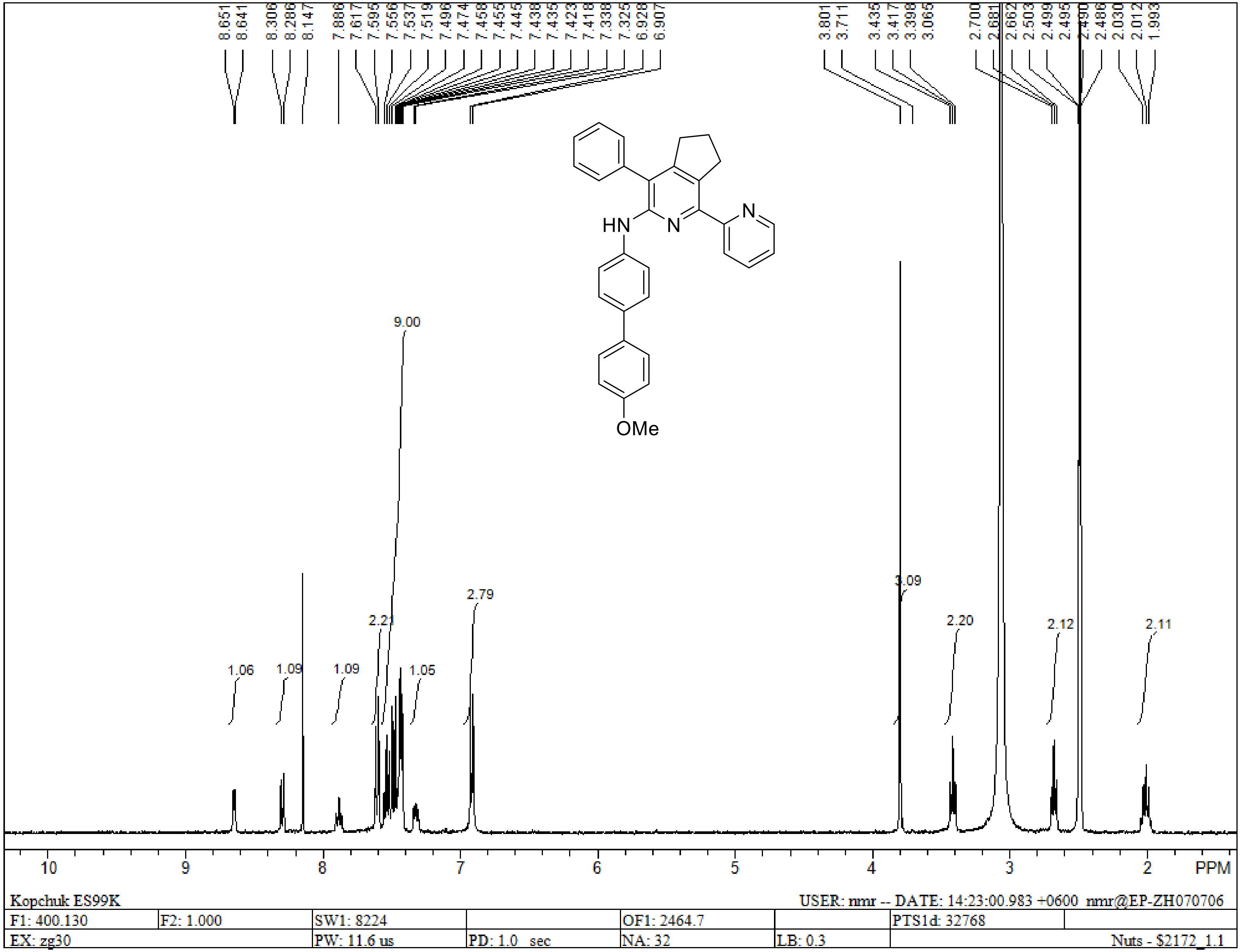

31. Figure S28. ^{13}**C NMR (400 MHz, DMSO-*d*$_6$) of 3k**

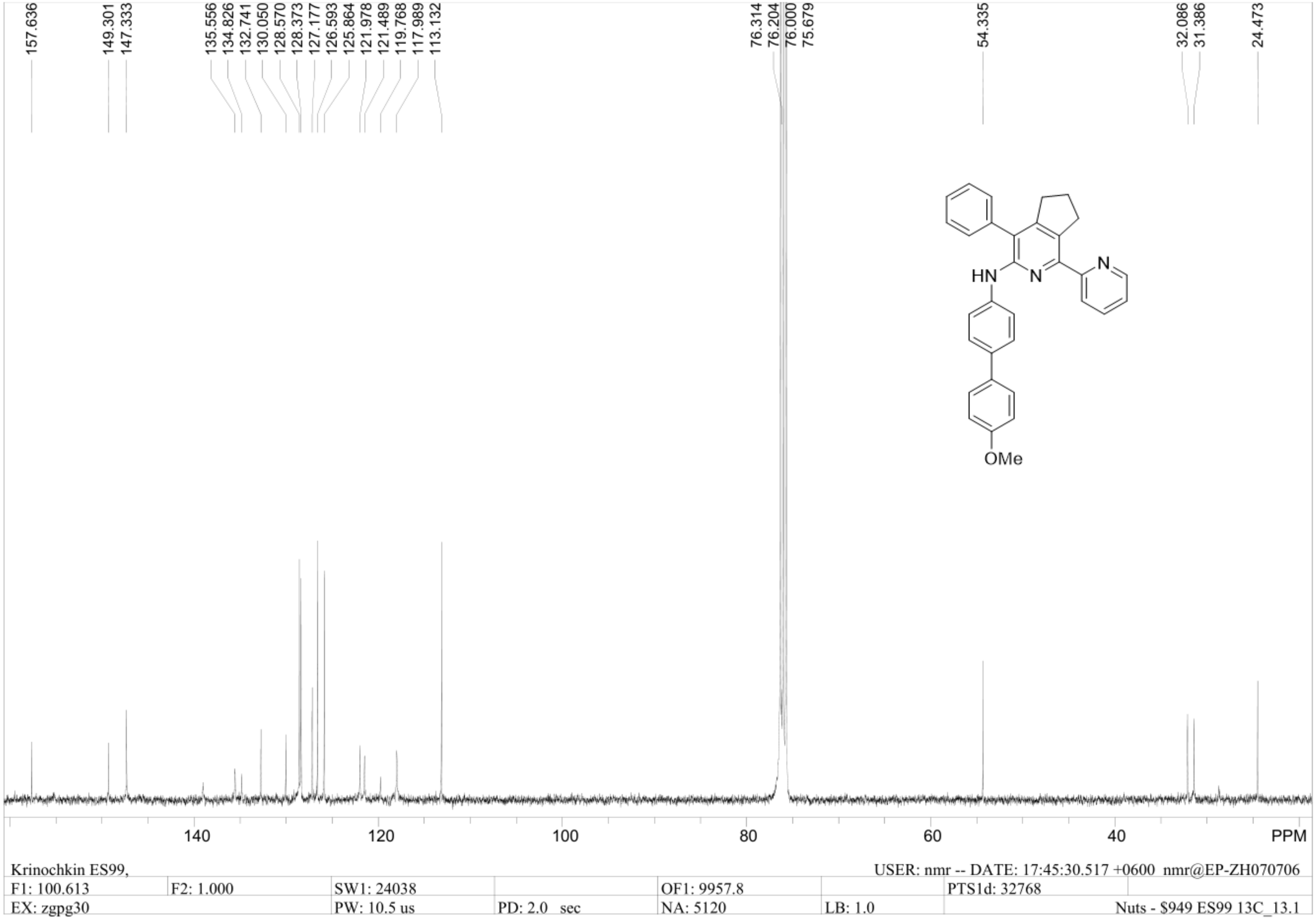

32. Figure S29.^{1}H NMR (400 MHz, DMSO-d_6) of 3l

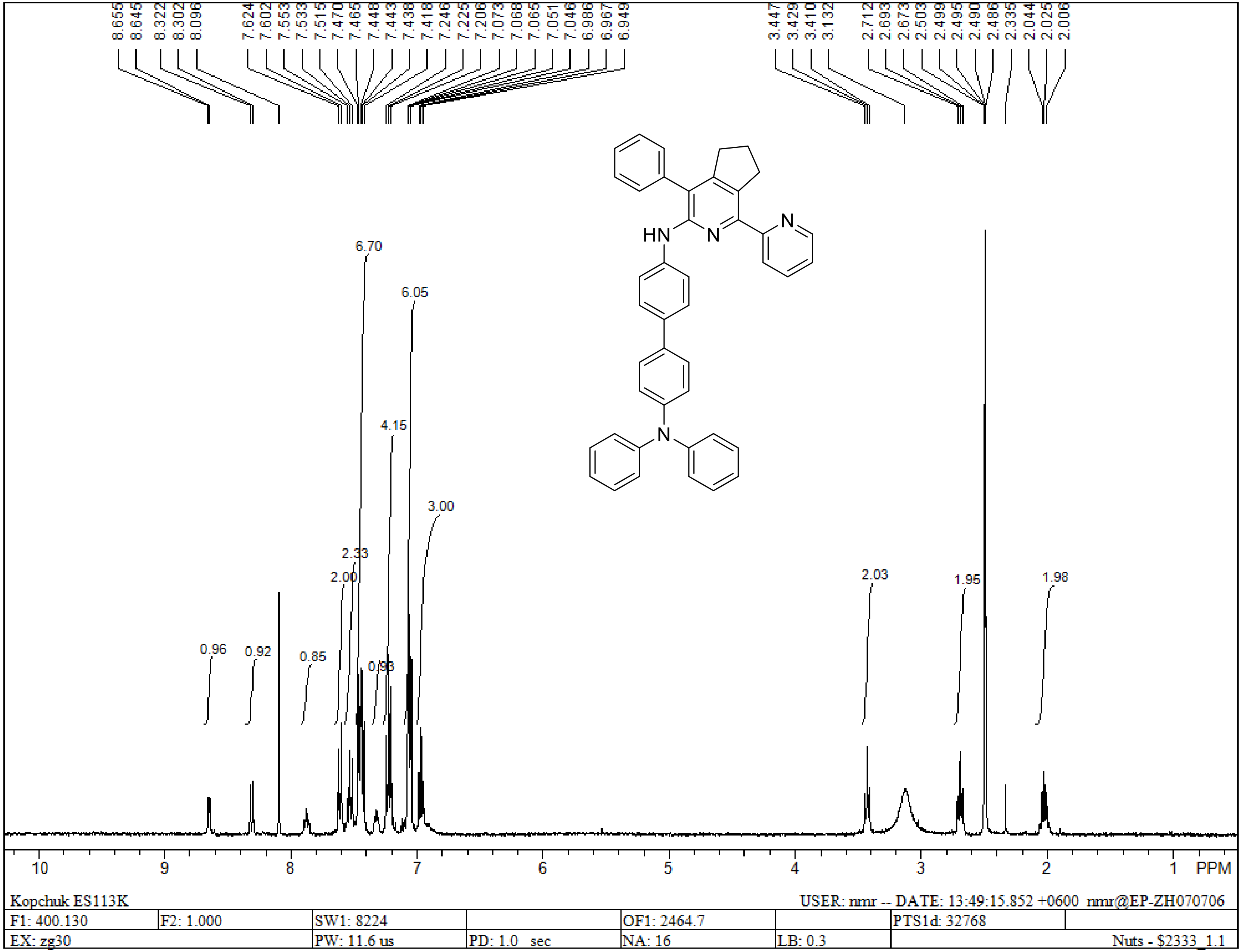

33. Figure S30.^{13}**C NMR (400 MHz, DMSO-**d_6**) of 3l**

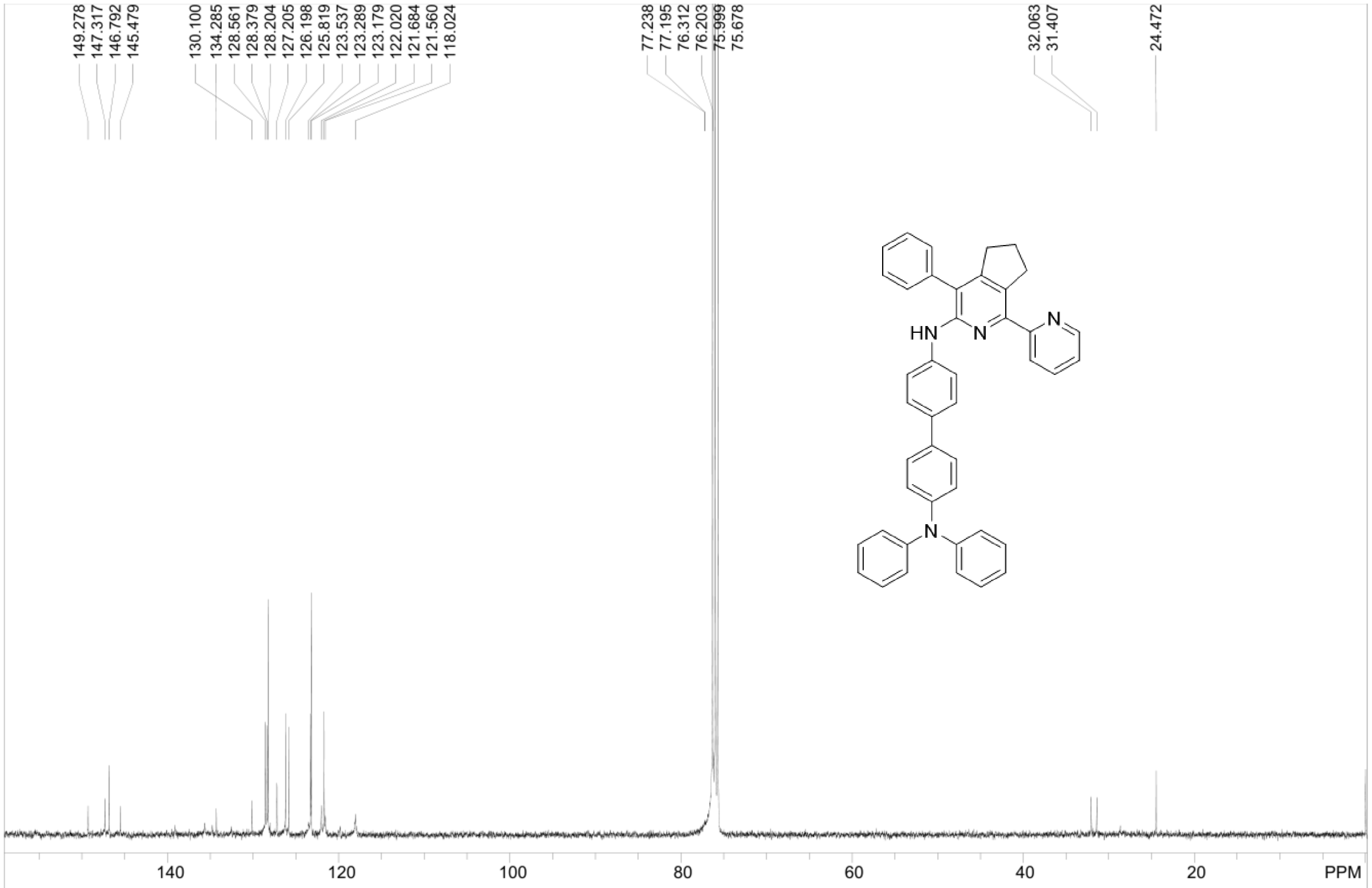

Krinochkin ES113,						USER: nmr -- DATE: 21:56:14.536 +0600 nmr@EP-ZH070706	
F1: 100.613	F2: 1.000	SW1: 24038		OF1: 9957.7		PTS1d: 32768	
EX: zgpg30		PW: 10.5 us	PD: 2.0 sec	NA: 5120	LB: 1.0		Nuts - $950 ES113 13C_13.1

34. Figure S31. Absorption and emission spectra of 3a-l and 5a-c in THF

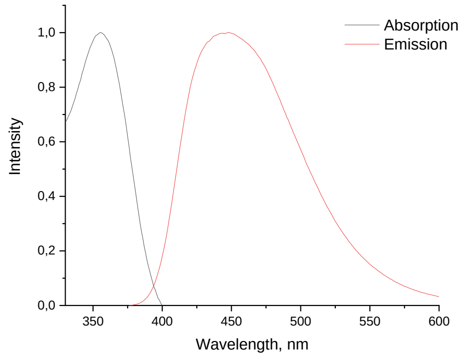

3a

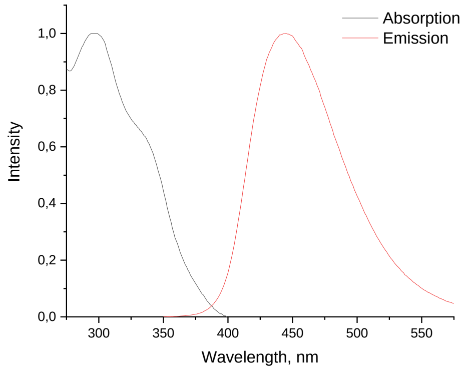

3b

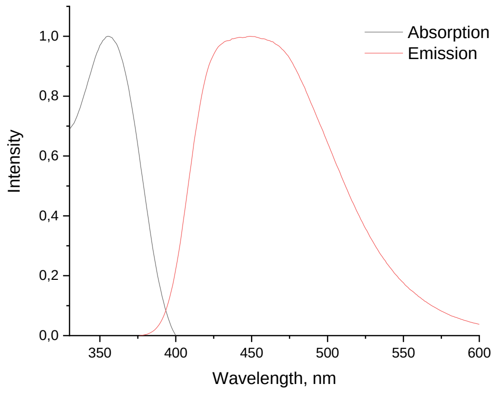

3c

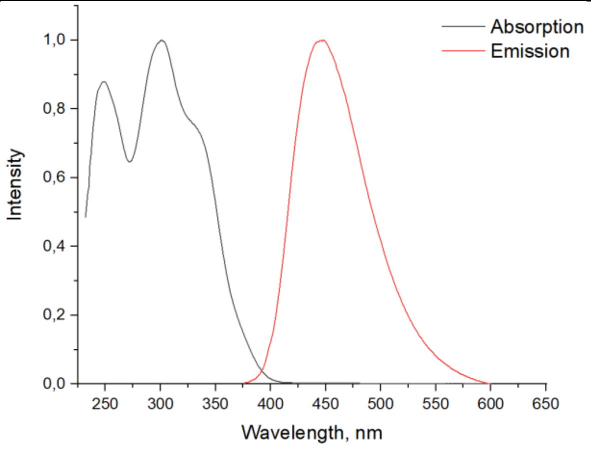

3d

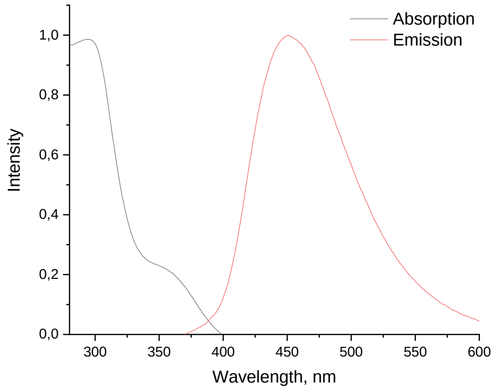

3e

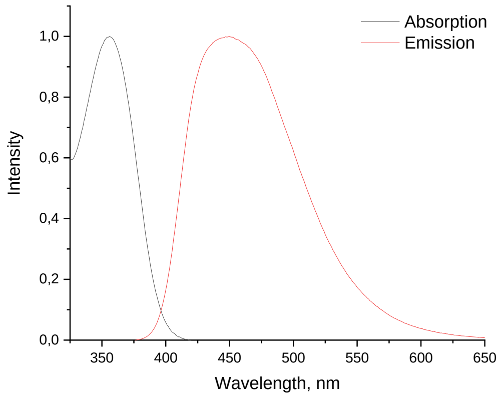

3f

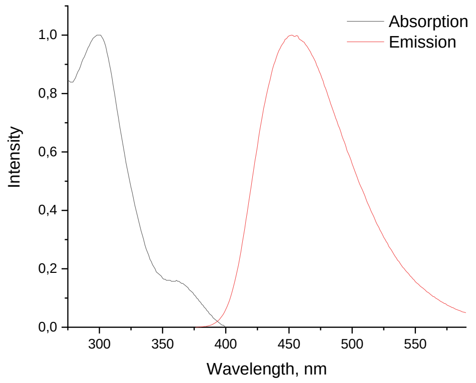

3g

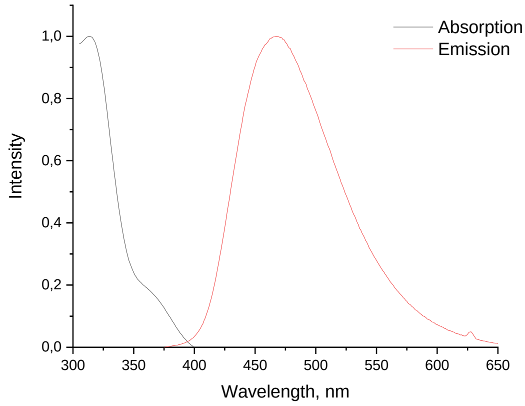

3h

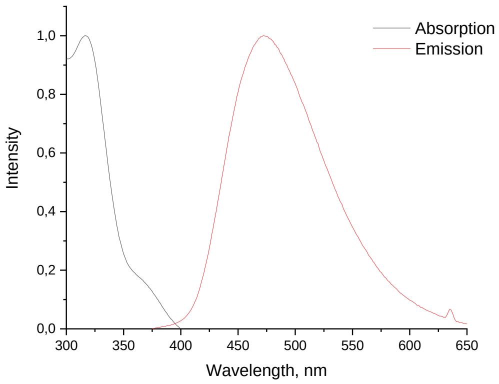

3i

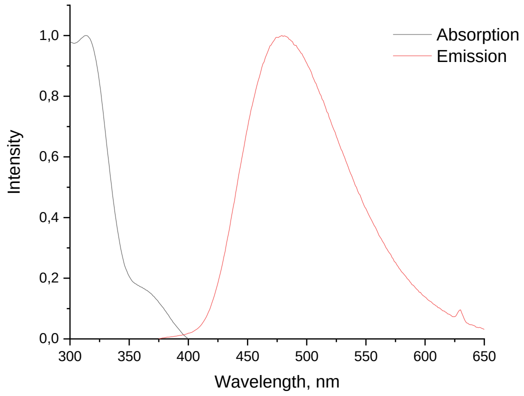

3j

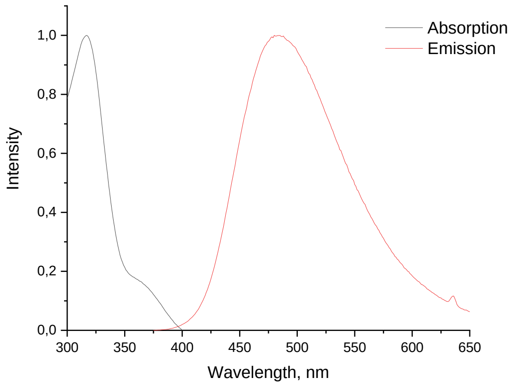

3k

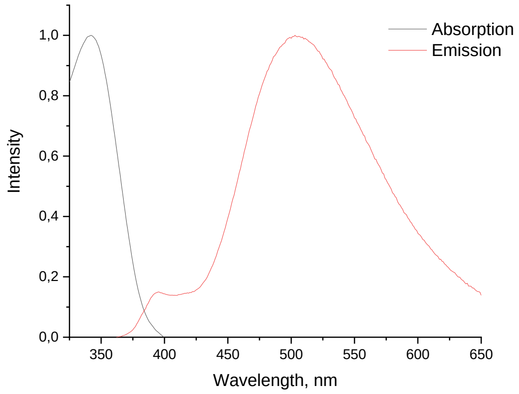

3l

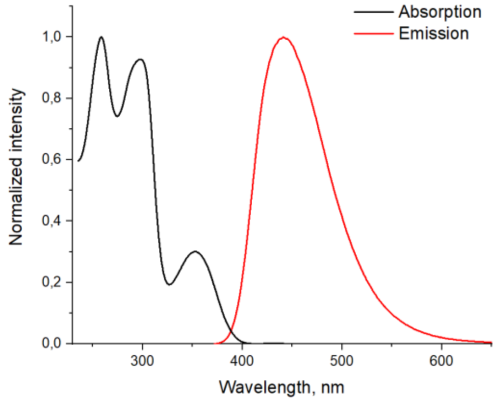
Absorption
Emission
Normalized intensity
1,0
0,8
0,6
0,4
0,2
0,0
300
400
500
600
Wavelength, nm

5a

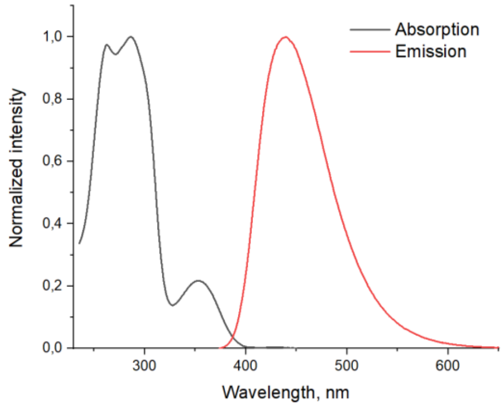
Absorption
Emission
Normalized intensity
1,0
0,8
0,6
0,4
0,2
0,0
300
400
500
600
Wavelength, nm

5b

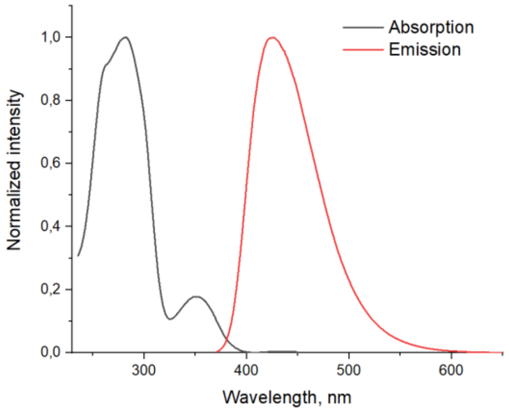
Absorption
Emission
Normalized intensity
1,0
0,8
0,6
0,4
0,2
0,0
300
400
500
600
Wavelength, nm

5c

35. **Table S2 Orientation polarizability for solvents (Δf), absorption and fluorescence emission maxima (λ_{abs}, λ_{em}, nm) and Stokes shift (nm, cm^{-1}) of 3b in different solvents**

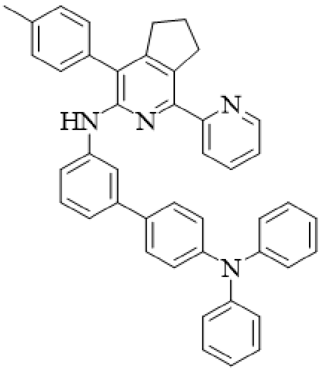

Solvent	Δf	λ_{abs}, nm	λ_{em}, nm	Stokes shift, nm	Stokes shift, cm^{-1}
n-Heptane	0.0001	310	409	99	7808
Cyclohexane	0.001	308	408	100	7958
Toluene	0.0126	313	424	111	8364
1,4-Dioxane	0.131	312	438	126	9220
Ethyl acetate	0.201	308	441	133	9792
THF	0.21	297	444	147	11148
DCM	0.22	295	438	143	11067
DMSO	0.26	284	478	194	14290
DMF	0.31	300	469	169	8981
Acetonitrile	0.3	323	455	132	12011
Methanol	0.31	307	410	103	8183

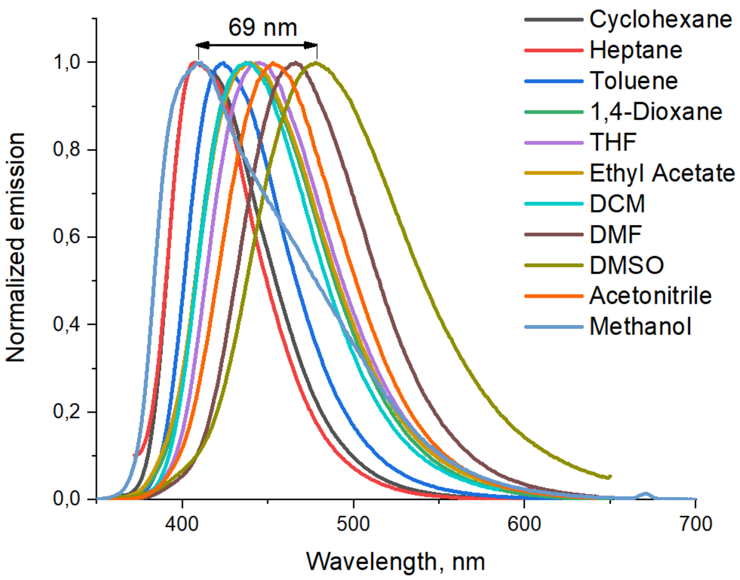

36. **Figure S32. Normalized fluorescence emission spectra of 3b in different solvents**

37. **Table S3 Orientation polarizability for solvents (Δf), absorption and fluorescence emission maxima (λ_{abs}, λ_{em}, nm) and Stokes shift (nm, cm^{-1}) of 3g in different solvents**

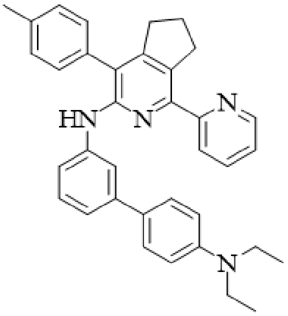

Solvent	Δf	λ_{abs}, nm	λ_{em}, nm	Stokes shift, nm	Stokes shift, cm^{-1}
n-Heptane	0.0001	300	413	113	9120
Cyclohexane	0.001	309	414	105	8208
Toluene	0.0126	308	431	123	9266
1,4-Dioxane	0.131	302	446	144	10691
Ethyl acetate	0.201	300	449	149	11062
THF	0.21	299	452	153	11321
DCM	0.22	300	449	149	11062
DMSO	0.26	297	480	183	12837
DMF	0.27	302	474	172	12016
Acetonitrile	0.3	315	465	150	10240
Methanol	0.31	302	437	135	10229

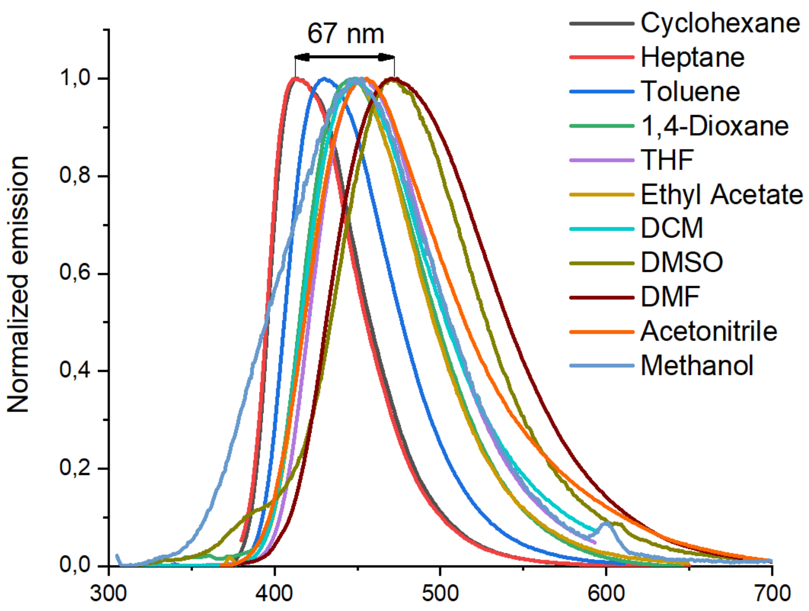

38. **Figure S33. Normalized fluorescence emission spectra of 3g in different solvents**

39. Table S4 Orientation polarizability for solvents (Δf), absorption and fluorescence emission maxima (λ_{abs}, λ_{em}, nm) and Stokes shift (nm, cm^{-1}) of 3i in different solvents

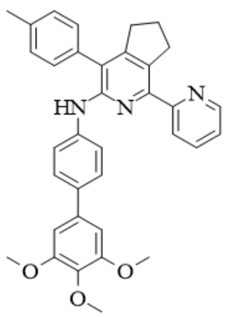

Solvent	Δf	λ_{abs}, nm	λ_{em}, nm	Stokes shift, nm	Stokes shift, cm^{-1}
n-Heptane	0.0001	315	418	103	7823
Cyclohexane	0.001	316	418	102	7722
Toluene	0.0126	322	440	118	8328
1,4-Dioxane	0.131	324	463	139	9266
THF	0.21	320	473	153	10108
Ethyl acetate	0.201	326	467	141	9262
DCM	0.22	318	464	146	9895
DMSO	0.26	323	515	192	11542
DMF	0.2746	322	497	175	10935
Acetonitrile	0.3	318	482	164	10700
Methanol	0.31	324	480	156	10030

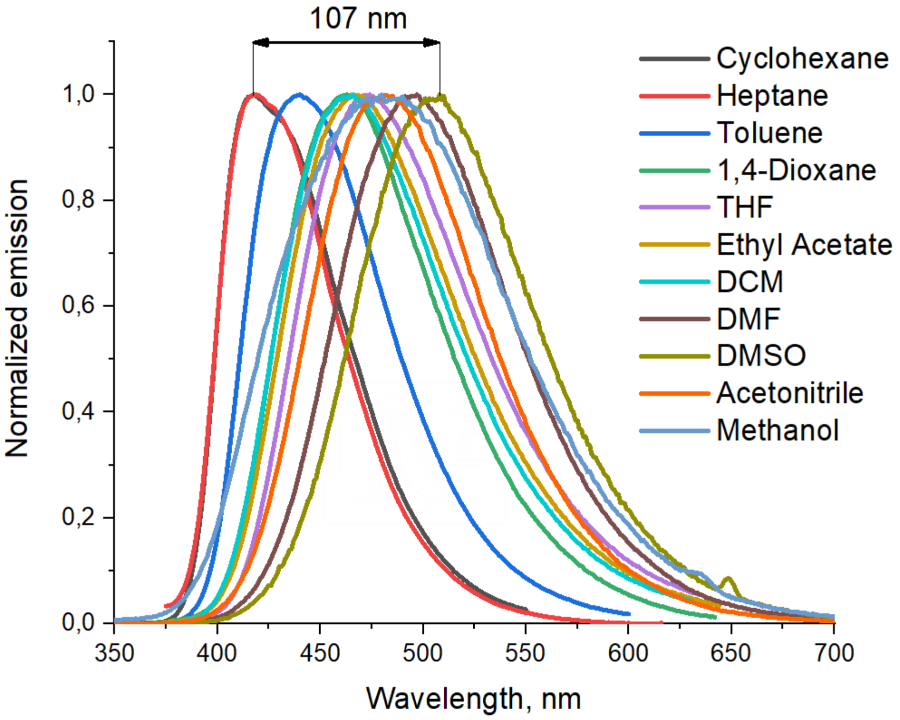

40. Figure S34. Normalized fluorescence emission spectra of 3i in different solvents

41. Table S5 Orientation polarizability for solvents (Δf), absorption and fluorescence emission maxima (λ_{abs}, λ_{em}, nm) and Stokes shift (nm, cm^{-1}) of 3l in different solvents

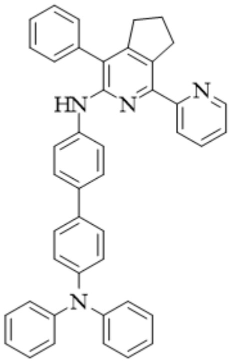

Solvent	Δf	λ_{abs}, nm	λ_{em}, nm	Stokes shift, nm	Stokes shift, cm^{-1}
n-Heptane	0.0001	338	441	103	6910
Cyclohexane	0.001	339	438	99	6667
Toluene	0.0126	342	463	121	7641
1,4-Dioxane	0.131	342	494	152	8997
Ethyl acetate	0.201	340	507	167	9688
THF	0.21	342	511	169	9670
DCM	0.22	343	519	176	9887
DMSO	0.26	336	418	82	5838
DMF	0.2746	339	418	79	5575
Acetonitrile	0.3	339	416	77	5460
Methanol	0.31	339	407	68	4928

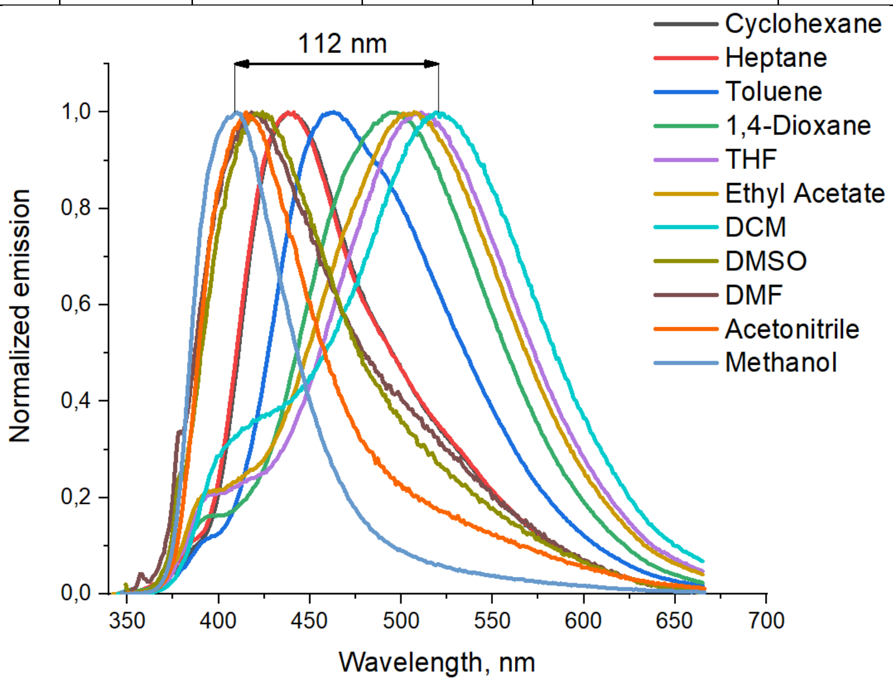

42. Figure S35 Normalized fluorescence emission spectra of 3l in different solvents

43. Table S6. Data for the Lippert-Mataga plots of 3b,g,i,l

#	3b	3g	3i	3l
**Equation**	y = a + b*x	y = a + b*x	y = a + b*x	y = a + b*x
**Intercept**	8867,87973 ± 231,41691	7928,46552 ± 275,62474	7934,9836 ± 179,83729	6817,00265 ± 272,53016
**Slope**	11193,71859 ± 1580,02017	12750,51884 ± 1881,85316	8872,22561 ± 1227,85556	12500,72306 ± 1860,72465
**Residual Sum of Squares**	808600,07418	1,14704E6	488318,38598	1,12143E6
**Pearson's r**	0,95363	0,94962	0,9553	0,94882
**R-Square (COD)**	0,90941	0,90178	0,91261	0,90027
**Adj. R-Square**	0,89129	0,88214	0,89513	0,88032

44. Table S7. Data for the Lippert-Mataga plots of 3b,g,i,l in the range of polar solvents (DMSO, DMF, acetonitrile, methanol)

#	3b	3g	3i	3l
**Equation**	y = a + b*x	y = a + b*x	y = a + b*x	y = a + b*x
**Intercept**	30352,36769 ± 2399,20429	45844,51115 ± 2360,46215	17417,91958 ± 3087,31116	9785,07301 ± 1412,78369
**Slope**	-65797,62587 ± 8364,30537	-122235,83001 ± 8229,23928	-22824,55324 ± 10763,2407	-15147,81417 ± 4925,36388
**Residual Sum of Squares**	220780,63332	213707,90927	365584,11738	76555,75735
**Pearson's r**	-0,98422	-0,9955	-0,83196	-0,90855
**R-Square (COD)**	0,96869	0,99102	0,69216	0,82546
**Adj. R-Square**	0,95304	0,98653	0,53825	0,73819

45. Table S8. Data for fluorescence emission maximum relationship with the empirical solvent polarity parameter $E_T(30)$ plots

#	3b	3g	3i	3l
**Equation**	y = a + b*x	y = a + b*x	y = a + b*x	y = a + b*x
**Intercept**	30509,80125 ± 1188,28148	30703,02139 ± 1199,34339	31227,47884 ± 1590,27638	35136,56307 ± 1671,95878
**Slope**	-202,8078 ± 30,83202	-219,13984 ± 31,11904	-250,55135 ± 41,26248	-394,87853 ± 46,95833

**Residual Sum of Squares**	1,98716E6	2,02433E6	3,5591E6	889940,81796
**Pearson's r**	-0,91867	-0,92795	-0,90648	-0,96642
**R-Square (COD)**	0,84396	0,86109	0,82171	0,93396
**Adj. R-Square**	0,82445	0,84372	0,79942	0,92075

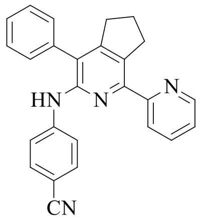

7.30 D

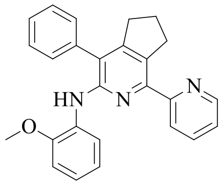

10.74 D

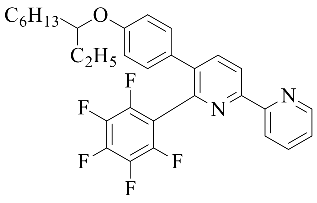

9.08 D

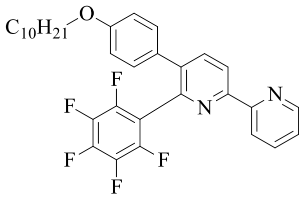

9.45 D

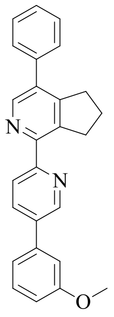

2.80 D

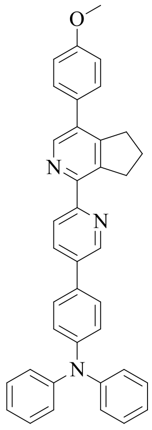

14.28 D

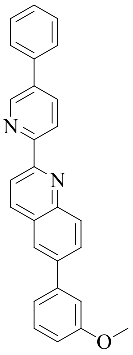

4.93 D

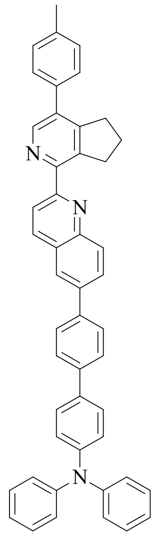

14.56 D

46. Figure S36. Difference in dipole moments (Δμ) of some 2,2’-bipyridine derivatives and analogs calculated according to Lippert-Mataga equation.

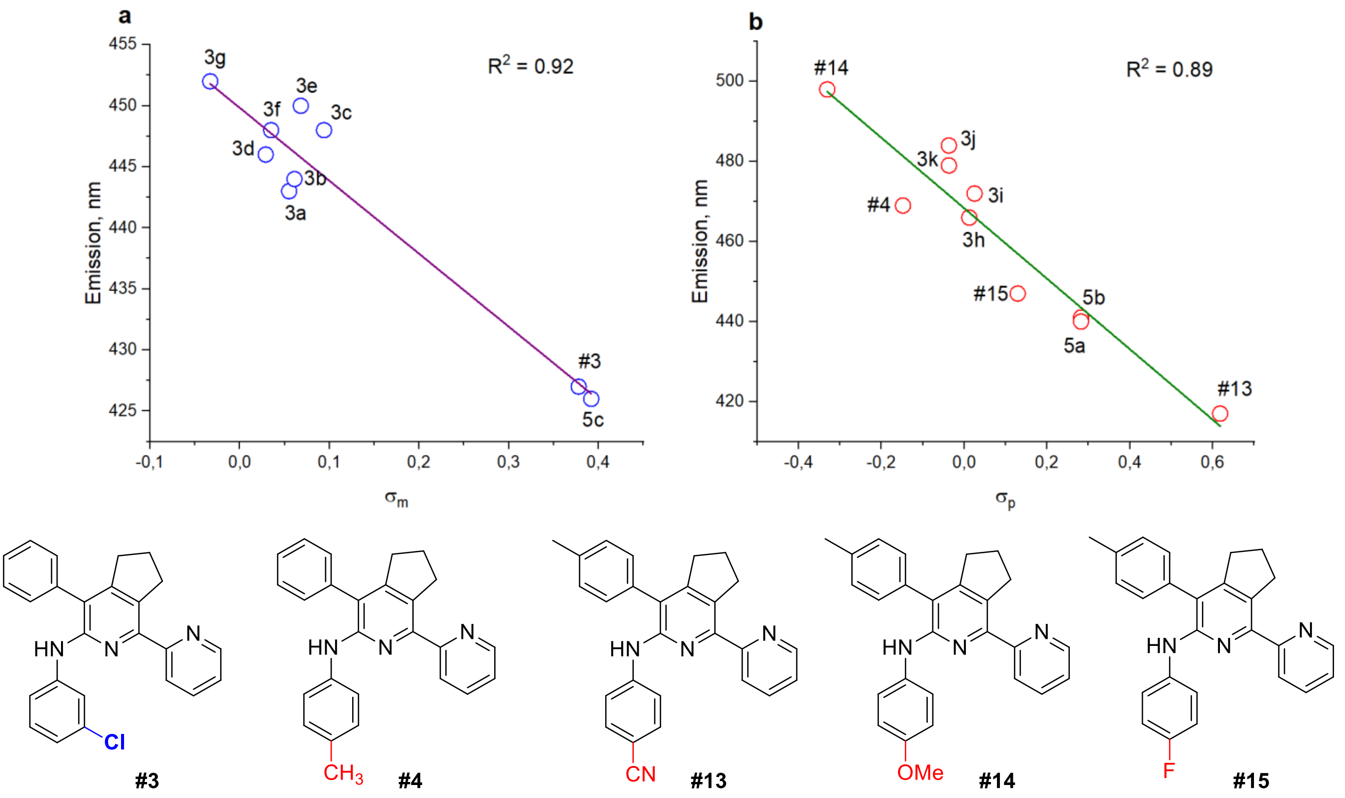

47. Figure S37. Plot of the Hammett constants at the meta (a) and para (b) position versus the values of the fluorophore emission maxima

Computational details

The quantum chemical calculations in ground (S_0) and excited (S_1) multiplicity states were carried out at the B3LYP/6-31G*//PM6 level of theory with the help of the Gaussian-09 [4] program package. No symmetry restrictions were applied during the geometry optimization procedure. The solvent effects were taken into account using the SMD (Solvation Model based on Density) continuum solvation model suggested by Truhlar and coworkers [5]. The Hessian matrices were calculated for all optimized model structures to prove the location of correct minima on the potential energy surface (no imaginary frequencies were found in all cases).

48. Table S9. Molecular electrostatic potential energy map and calculated dipole moments (in Debye) in ground (S_0) and excited (S_1) multiplicity states for 3b,g,i,l.

Model structures	MEP	S_0	S_1	Δμ
**3b**	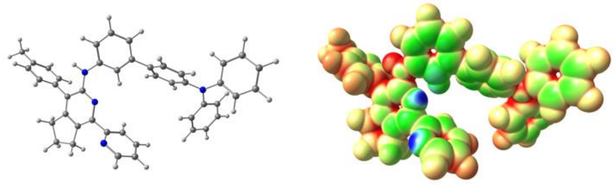	3.701	9.279	5.578

**3g**	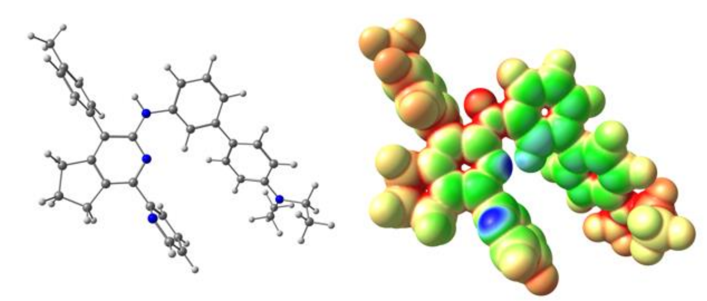	3.874	6.641	2.766
**3i**	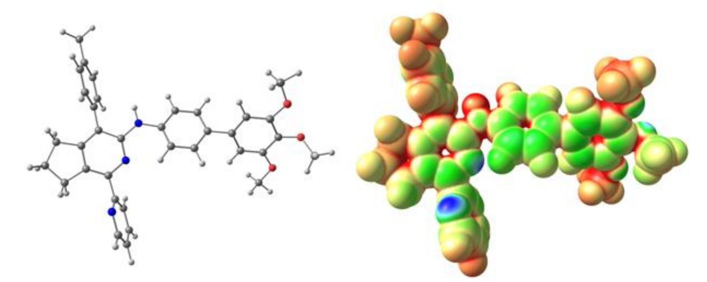	6.090	7.576	1.485
**3l**	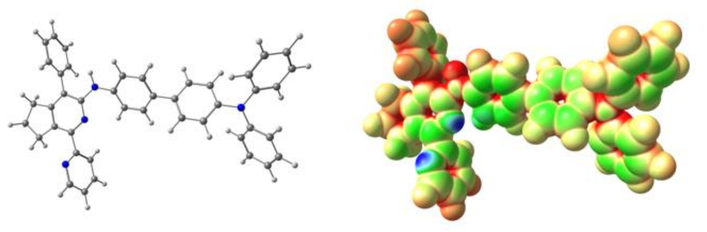	3.631	16.745	13.116

49. Table S10. HOMO-LUMO Energy levels for fluorophores 3b,g,i,l in a gas phase

	HOMO energy, eV	LUMO energy, eV	Gap, eV[a]	Gap, eV[b]
**3b**	-4.90	-0.84	4.06	4.50
**3g**	-4.75	-0.81	3.94	4.57
**3i**	-5.06	-0.83	4.23	4.55
**3l**	-4.82	-0.88	3.94	4.44

[a] According to the computational studies

[b] According to the UV/Vis data (**Figure S38**)

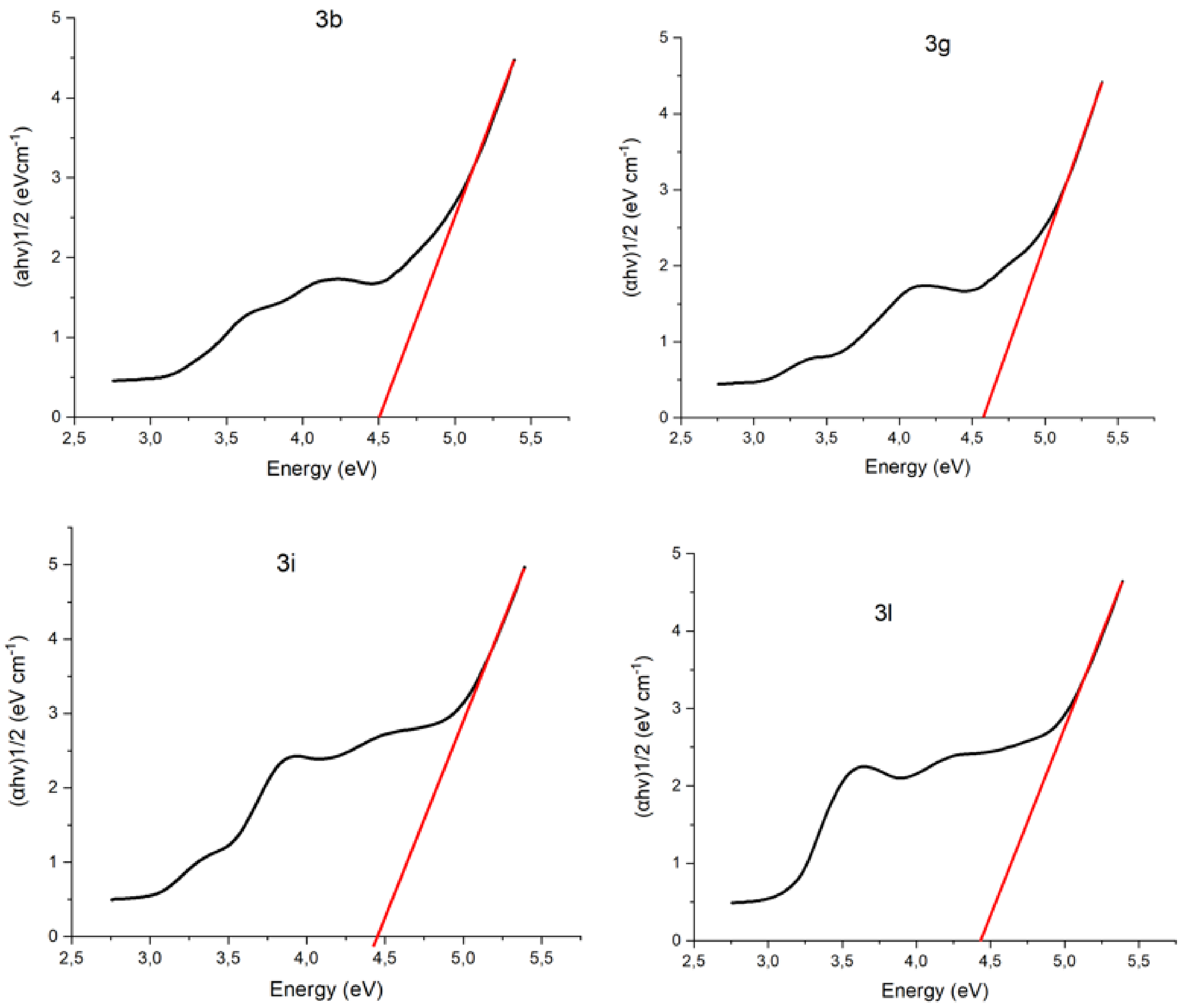

50. Figure S38. Tauc Plots for 3b,g,i,l constructed based on UV/Vis data

References

[1] Kozhevnikov, V.N.; Kozhevnikov, D.N.; Nikitina, T. v.; Rusinov, V.L.; Chupakhin, O.N.; Zabel, M.; König, B. A Versatile Strategy for the Synthesis of Functionalized 2,2′-Bi- and 2,2′:6′,2″-Terpyridines *via* Their 1,2,4-Triazine Analogues. *J. Org. Chem.* **2003**, *68*, 2882–2888. https://doi.org/10.1021/jo0267955.

[2] Dolomanov, O.V.; Bourhis, L.J.; Gildea, R.J.; Howard, J.A.K.; Puschmann, H. *OLEX2*: A Complete Structure Solution, Refinement and Analysis Program. *J. Appl. Crystallogr.* **2009**, *42*, 339–341. https://doi.org/10.1107/S0021889808042726.

[3] Sheldrick, G.M. *SHELXT*—Integrated Space-Group and Crystal-Structure Determination. *Acta Crystallogr. A Found. Adv.* **2015**, *71*, 3–8. https://doi.org/10.1107/S2053273314026370.

[4] Frisch, M.J.; Trucks, G.W.; Schlegel, H.B.; Scuseria, G.E.; Robb, M.A.; Cheeseman, J.R.; Scalmani, G.; Barone, V.; Petersson, G.A.; Nakatsuji, H.; et al. *Gaussian 09, Revision C.01*; Gaussian, Inc.: Wallingford, CT, USA, 2010.

[5] Marenich, A.V.; Cramer, C.J.; Truhlar, D.G.; Universal Solvation Model Based on Solute Electron Density and on a Continuum Model of the Solvent Defined by the Bulk Dielectric Constant and Atomic Surface Tensions. *J. Phys. Chem. B*, **2009**, *113*, 6378. https://doi.org/10.1021/jp810292n.
